# A Combined Experimental and Theoretical Investigation of Oxidation Catalysis by *cis*-[V^IV^(O)(Cl/F)(N_4_)]^+^ Species Mimicking the Active Center of Metal-Enzymes

**DOI:** 10.1021/acs.inorgchem.2c02526

**Published:** 2022-11-10

**Authors:** Michael G. Papanikolaou, Anastasia V. Simaioforidou, Chryssoula Drouza, Athanassios C. Tsipis, Haralampos N. Miras, Anastasios D. Keramidas, Maria Louloudi, Themistoklis A. Kabanos

**Affiliations:** † Section of Inorganic and Analytical Chemistry, Department of Chemistry, University of Ioannina, Ioannina45110, Greece; § Department of Agricultural Production, Biotechnology and Food Science, Cyprus University of Technology, 3036Limassol, Cyprus; ⊥ West CHEM, School of Chemistry, University of Glasgow, GlasgowG12 8QQ, U.K.; ∥ Department of Chemistry, University of Cyprus, Nicosia1678, Cyprus

## Abstract

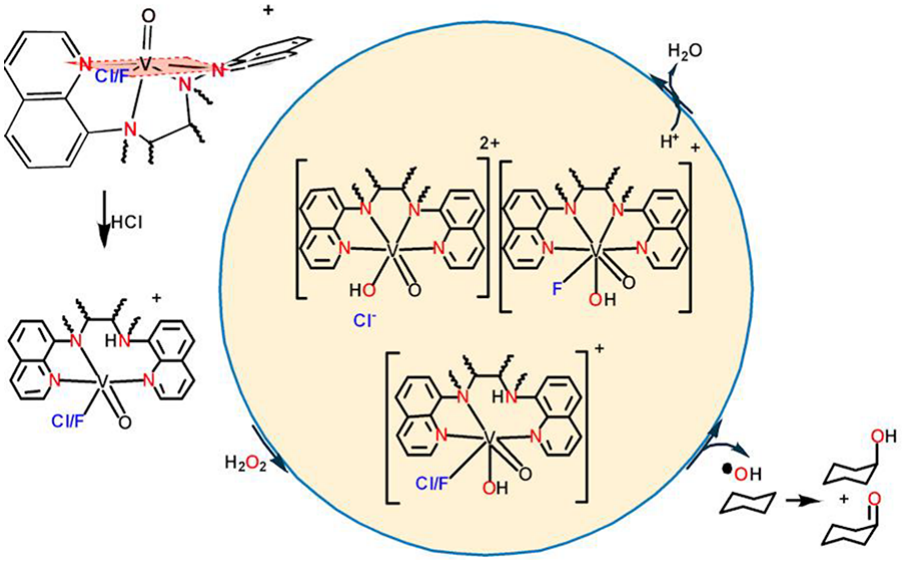

Reaction of V^IV^OCl_2_ with the nonplanar tetradentate N_4_ bis-quinoline ligands yielded four oxidovanadium(IV) compounds of the general formula *cis*-[V^IV^(O)(Cl)(N_4_)]Cl. Sequential treatment of the two nonmethylated N_4_ oxidovanadium(IV) compounds with KF and NaClO_4_ resulted in the isolation of the species with the general formula *cis*-[V^IV^(O)(F)(N_4_)]ClO_4_. In marked contrast, the methylated N_4_ oxidovanadium(IV) derivatives are inert toward KF reaction due to steric hindrance, as evidenced by EPR and theoretical calculations. The oxidovanadium(IV) compounds were characterized by single-crystal X-ray structure analysis, cw EPR spectroscopy, and magnetic susceptibility. The crystallographic characterization showed that the vanadium compounds have a highly distorted octahedral coordination environment and the *d*(V^IV^–F) = 1.834(1) Å is the shortest to be reported for (oxido)(fluorido)vanadium(IV) compounds. The experimental EPR parameters of the V^IV^O^2+^ species deviate from the ones calculated by the empirical additivity relationship and can be attributed to the axial donor atom trans to the oxido group and the distorted V^IV^ coordination environment. The vanadium compounds act as catalysts toward alkane oxidation by aqueous H_2_O_2_ with moderate ΤΟΝ up to 293 and product yields of up to 29% (based on alkane); the vanadium(IV) is oxidized to vanadium(V), and the ligands remain bound to the vanadium atom during the catalysis, as determined by ^51^V and ^1^H NMR spectroscopies. The cw X-band EPR studies proved that the mechanism of the catalytic reaction is through hydroxyl radicals. The chloride substitution reaction in the *cis*-[V^IV^(O)(Cl)(N_4_)]^+^ species by fluoride and the mechanism of the alkane oxidation were studied by DFT calculations.

## Abstract 2

Highly distorted N_4_−oxidovanadium(IV), *cis*-[V^IV^(=O)(Cl/F)(N_4_)]^+^, compounds mimicking the distorted coordination environment of the metal-ion in metal-enzymes’ active center catalyze oxidation of alkanes. The structural distortion results in significant deviation of the cw EPR parameters from the expected ones and equilibrium between six- and five- coordinate species in solution. DFT studies show that distortion, steric hindrance, and the coordinated halogen define the catalytic mechanism of oxidation of alkanes by *cis*-[V^IV^(=O)(Cl/F)(N_4_)]^+^ compounds.

## Introduction

In recent years, the coordination chemistry of vanadium has drawn a lot of interest, mainly due to its biological, medicinal and catalytic applications.^1−11^ Vanadium exhibits a wide variety of oxidation states (−III to + V), with the oxidation states of +III to +V mainly found in molecular systems of biological relevance. Enzymes, such as the vanadium-dependent haloperoxidases found in algae, are able to utilize vanadium’s wide range of oxidation states in order to oxidize halides in nature.^12^ Moreover, vanadium plays a key role in the vanadium nitrogenase enzyme, which is a vanadium analogue of the iron–molybdenum enzyme that reduces dinitrogen to ammonia.^13−15^ In addition, vanadium has a significant effect on cell growth, signaling processes, antitumor activity, and insulin-mimetic properties.^16−26^

The synthesis of metal compounds, which are metal enzymes’ active site analogues, has played an important role in understanding the mechanisms of enzyme activity and in the development of small molecules with activity similar to relevant enzymes.^27^ The particular function of the metal-enzymes requires specific oxidation states, ligands, and coordination geometries for the metal-ion in the active site.^27^ The coordination geometries around the metal-ions in the enzymes’ active site, enforced by the rigidity of the protein backbones, are irregular. These enforced geometries define the activity of the enzyme.^28,29^ Small changes of these structural features are crucial for the specificity of the enzymes. In contrast, in the small metal compounds, there are few or no constraints dictating the geometry. Therefore, the arrangement of the ligands around the metal ions in these compounds relies on the preference of the metal ion.

Vanadium’s low-molecular-weight coordination compounds mimic the activity of enzymes, such as haloperoxidases.^30−33^ Vanadium’s wide range of oxidation states and coordination numbers and its Lewis acid character are the key characteristics that enable the use of vanadium compounds in various catalytic reactions, mimicking haloperoxidases, such as alcohol oxidation, sulfoxidation, epoxidation, and alkane oxidation reactions.^34−41^ In particular, the oxidation of alkanes has a high industrial significance, since it enables the functionalization of inert alkanes to more valuable and reactive organic materials such as alcohols and ketones in the presence of a suitable oxidant like H_2_O_2_ or O_2_, under mild conditions.^42−45^ Some of the most commonly used ligands are nitrogen-based tetradentate pyridine or quinoline ligands, which have the ability to strongly bind and stabilize vanadium ions in the +IV or +V oxidation state. Moreover, these ligands are highly resistant to oxidation and decomposition under the catalytic conditions, and their compounds with iron(II) are some of the most efficient catalysts for alkane oxidation.^46^ However, in order to mimic metal-enzymes outstanding oxidative activity and synthesize efficient low-molecular-weight catalysts, it is important to understand the effect of the distortion of the coordination environments of the metal ions in the active site of the enzyme and its contribution to the catalytic action.

**Scheme 1:**
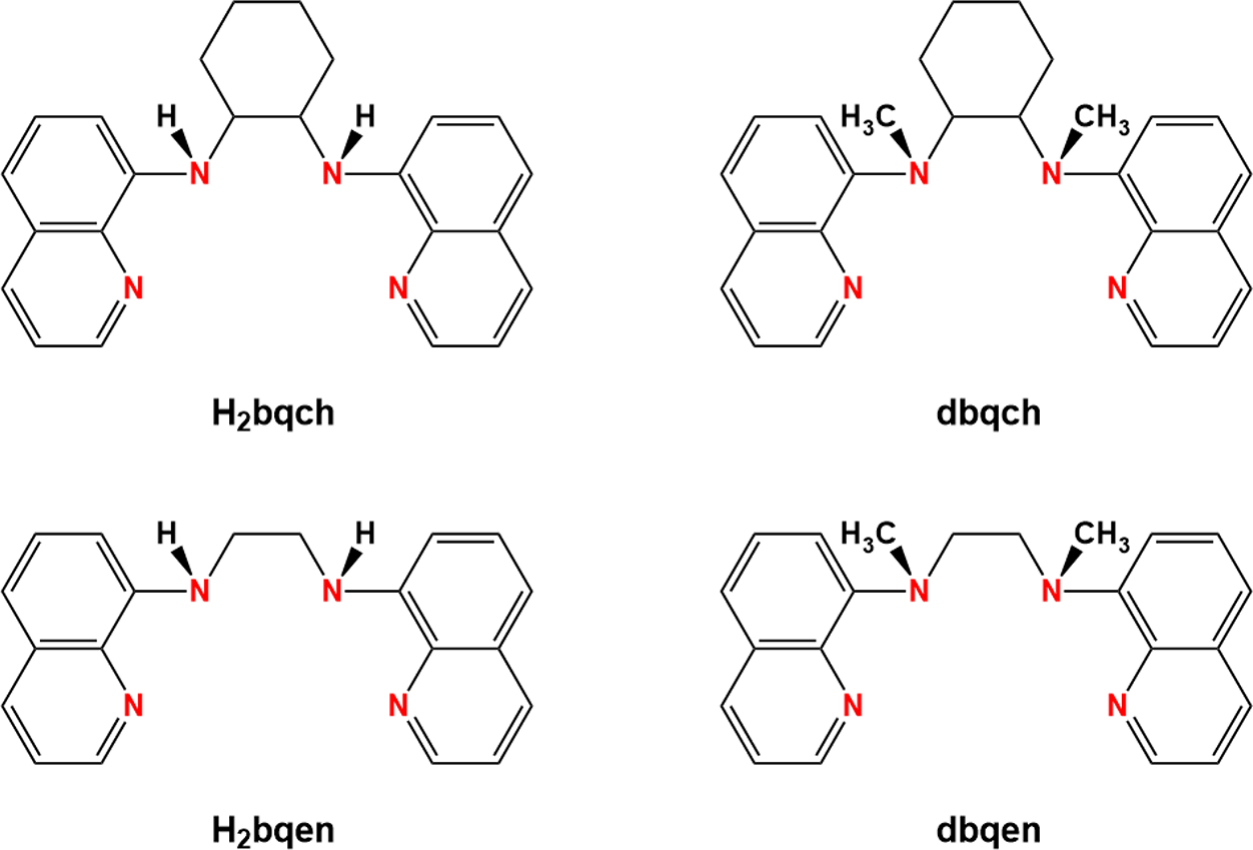
Drawing of the Ligands Used in This Study

Herein, we describe the synthesis, physicochemical, and structural characterization and the catalytic properties in alkane oxidation reactions of various oxidovanadium(IV) compounds with the nonplanar N_4_ quinoline-based/amine ligands and their dimethylated analogues (Scheme 1).

**Scheme 2:**
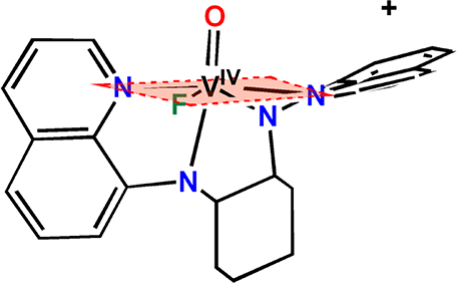
Highly Distorted Equatorial Plane of the Octahedral Compound *cis*-[V^IV^(O)(F)(N_4_)]^+^

The tetradentate nonplanar N_4_ ligands (Scheme 1) were chosen because their ligation to V^IV^O^2+^ induces a severely distorted octahedral geometry (Scheme 2), since our aim was to study the effect of structural distortions on the catalytic properties and the substitution reactions of these *cis*-[V^IV^(O)(X)(N_4_)]^+^ species. The geometric features of these *cis*-[V^IV^(O)(X)(N_4_)]^+^ (X = F^–^, Cl^–^) species mimic the irregularities of the enzymes’ active site, and can for the first time provide valuable information regarding their impact on the oxidative catalytic activity, the mechanism of their action (DFT calculations), and their spectroscopic properties (cw X-band EPR). Moreover, the effect of the halogen, in the *cis*-[V^IV^(O)(X)(N_4_)]^+^ (X = F^–^, Cl^–^) species, on their oxidative catalytic activity and the catalytic mechanism (DFT calculations) was also investigated.

## Experimental Section

### Synthesis of the Ligands and the Oxidovanadium(IV) Compounds

#### *N*,*N*′-Bis(8-quinolyl)cyclohexane-1,2-diamine, (H_2_bqch)

*trans*-Diaminocyclohexane (2.40 mL, 2.28 g, 20 mmol) and sodium metabisulfite (7.60 g, 40 mmol) were added to a suspension of 8-hydroxyquinoline (5.80 g, 40 mmol) in 200 mL of water. The mixture was heated at reflux for 10 days. Subsequently, the solution was cooled to room temperature and was made strongly alkaline (pH 13) with the addition of solid KOH. The mixture was extracted with dichloromethane (3 × 40 mL), the organic layers were combined, dried with MgSO_4_, and the solvent was removed under vacuum. The obtained residue was triturated with warm (40 °C) ethyl alcohol (10 mL), and the formed pale-yellow precipitate was filtered and dried under high vacuum to get 2.95 g of the desired organic molecule. Yield, 40%, based on *trans*-diaminocyclohexane. Anal. Calcd (%) for C_24_H_24_N_4_ (*M*_r_ = 368.24 g/mol): C, 78.22; H, 6.57; N, 15.21. Found (%): C, 78.20; H, 6.58; N, 15.28. *R*_f_ = 0.84 (CH_3_COOC_2_H_5_). Mp = 174–175 °C.

#### *N*,*N*′-Dimethyl-*N*,*N*′-bis(8-quinolyl)cyclohexane-1,2-diamine, (dbqch)

The dimethylated organic molecule dbqch was prepared according to Britovsek and co-workers in 61% yield.^46^ The purity of dbqch was confirmed with positive HR-ESI-MS, and ^1^H, ^13^C NMR. Anal. Calcd (%) for C_26_H_28_N_4_ (*M*_r_ = 396.28 g/mol): C, 78.74; H, 7.12; N, 14.14. Found (%): C, 78.53; H, 7.08; N, 14.28.

#### *N,N*′-Bis(8-quinolyl)ethane-1,2-diamine (H_2_bqen)

This organic molecule was synthesized in the same way as H_2_bqch, except that ethylene-1,2-diamine (1.40 mL, 1.20 g, 20 mmol) was used instead of *trans*-diaminocyclohexane. The final product was a yellow solid (3.77 g, 60% based on ethylene-1,2-diamine). Anal. Calcd (%) for C_20_H_18_N_4_ (*M*_r_ = 314.19 g/mol): C, 76.39; H, 5.77; N, 17.83. Found (%): C, 76.35; H, 5.71; N, 17.84. *R*_f_ = 0.81 (CH_3_COOC_2_H_5_). Mp = 160–161 °C.

#### *N*,*N*′-Dimethyl-*N,N*′-bis(8-quinolyl)ethane-1,2-diamine (dbqen)

The dimethylated organic molecule H_2_bqch was prepared according to Britovsek and co-workers in 69% yield.^46^ The purity of dbqen was confirmed with positive HR-ESI-MS, and ^1^H, ^13^C NMR. Anal. Calcd (%) for C_22_H_22_N_4_ (*M*_r_ = 342.22 g/mol): C, 77.15; H, 6.48; N, 16.37. Found (%): C, 76.215; H, 6.44; N, 16.18.

#### *Cis*-chlorido[*N*,*N*′-Bis(8-quinolyl)cyclohexane-1,2-diamine-*N*,*N*,*N*,*N*]oxidovanadium(IV) Chloride, *cis*-[V^IV^(O)(Cl)(H_2_bqch)]Cl·H_2_O (**1**·H_2_O)

To the stirred aqueous solution (5 mL) of V^IV^OSO_4_·5H_2_O (172 mg, 0.68 mmol), BaCl_2_·2H_2_O (183 mg, 0.75 mmol) was added in one portion, and a white precipitate (BaSO_4_) was immediately formed. The mixture was stirred for 1 h and was filtered. The filtrate was evaporated to dryness under high vacuum, and the residue was dissolved in CH_3_CN (6 mL). A tetrahydrofuran (20 mL) solution containing the ligand H_2_bqch (250 mg, 0.68 mmol) was added dropwise to the stirred oxidovanadium(IV) solution. Upon addition of the ligand, the blue color of the solution changed to brown, and a light brown precipitate was formed. The solution was stirred for three additional hours, and then it was filtered and washed with diethyl ether (2 × 10 mL) and dried in vacuum to get 0.275 g of a light brown solid. Yield, 81% (based on H_2_bqch). Anal. Calcd (%) for C_24_H_26_Cl_2_N_4_O_2_V (*M*_r_= 524.10 g/mol): C, 54.96; H, 5.00; Cl, 13.53; N, 10.69; V, 9.72. Found (%): C, 54.91; H, 4.87; Cl, 13.47; N, 10.75; V, 9.67. (High resolution electrospray ionization mass spectrometry [HR-ESI(+)-MS]: calcd for *cis*-[V^IV^(O)(Cl)(H_2_bqch)]Cl·H_2_O (C_24_H_26_Cl_2_N_4_O_2_V) {[M-(Cl+H_2_O)]^+^} *m*/*z* 470.1073, found 470.1075 . μ_eff_ = 1.73 μ_B_

#### *Cis*-chlorido[*N*,*N*′-dimethyl-*N*,*N*′-bis(8-quinolyl)cyclohexane-1,2-diamine-*N*,*N*,*N*,*N*]oxidovanadium(IV) Chloride, *cis*-[V^IV^(O)(Cl)(dbqch)]Cl (**2**)

Compound **2** was synthesized using the same method reported for **1·**H_2_O with V^IV^OSO_4_·5H_2_O (96 mg, 0.38 mmol, 1 equiv) and dbqch (150 mg, 0.38 mmol, 1 equiv). Yield: 120 mg (59%) of a green solid. Anal. Calcd (%) for C_26_H_28_Cl_2_N_4_OV (*M*_r_ = 534.12 g/mol): C, 58.42; H, 5.28; Cl, 13.28; N, 10.49; V, 9.53. Found (%): C, 58.47; H, 4.97; Cl, 13.19; N, 10.68; V, 9.28. (High resolution electrospray ionization mass spectrometry [HR-ESI(+)-MS]: calcd for C_26_H_28_Cl_2_N_4_OV {[M-(Cl)]^+^} *m*/*z* 498.1386, found 498.1368. μ_eff_ = 1.71 μ_B_

Crystals of *cis*-[V^IV^(O)(Cl)(dbqch)]BF_4_·2CH_3_CN (**2’**) suitable for X-ray structure analysis were prepared as follows: Compound **2** (50 mg, 0.09 mmol) was dissolved in water (10 mL) under magnetic stirring, and NH_4_BF_4_ (9.8 mg, 0.09 mmol) was added to it. Upon addition of NH_4_BF_4_, a light green precipitate was formed which was filtered and dried. Dissolution of the green solid in CH_3_CN and layering of diethyl ether to it resulted in the formation of crystals of *cis*-[V^IV^(O)(Cl)(dbqch)]BF_4_·2CH_3_CN.

Crystals of *cis*-[V^IV^(O)(Cl)(dbqch)]ClO_4_ (**2’’**) suitable for X-ray structure analysis were prepared using the same method reported for **2’** except that NaClO_4_ was used instead of NH_4_BF_4_.

**Caution!***Perchlorates are powerful oxidizers, they are potentially hazardous, especially in contact with reducing material, and they may explode when exposed to shock or heat.*(47)

#### *Cis*-chlorido[*N,N*′-Bis(8-quinolyl)ethane-1,2-diamine-*N*,*N*,*N*,*N*]oxidovanadium(IV) Chloride, *cis*-[V^IV^(O)(Cl)(H_2_bqen)]Cl·2H_2_O (**3**·2H_2_O)

Compound **3·**2H_2_O was prepared using the same method reported for **1·**H_2_O with V^IV^OSO_4_·5H_2_O (240 mg, 0.95 mmol, 1 equiv), BaCl_2_·2H_2_O (256 mg, 1.05 mmol) and H_2_bqen (300 mg, 0.95 mmol, 1 equiv). Yield, 0.368 g (85%, based on H_2_bqen) of a brown solid. Anal. Calcd (%) for C_20_H_22_Cl_2_N_4_O_3_V (*M*_r_ = 488.07 g/mol): C, 49.18; H, 4.54; Cl, 14.53; N, 11.48; V, 10.44. Found (%): C, 49.23; H, 4.56; Cl, 14.49; N, 11.24; V, 10.31 [HR-ESI(+)-MS]: calcd for *cis*-[V^IV^(O)(Cl)(H_2_bqen)]Cl·2H_2_O (C_20_H_22_Cl_2_N_4_O_3_V) {[M-(Cl+H_2_O)]^+^} *m*/*z* 416.0603, found 416.0600. μ_eff_ = 1.70 μ_B_

#### *Cis*-chlorido[*N*,*N*′-dimethyl-*N,N*′-Bis(8-quinolyl)ethane-1,2-diamine-*N*,*N*,*N*,*N*]oxidovanadium(IV) Chloride, *cis*-[V^IV^(O)(Cl)(dbqen)]Cl·3H_2_O (**4**·3H_2_O)

Compound **4·**3H_2_O was prepared using the same method reported for **1·**H_2_O with V^IV^OSO_4_·5H_2_O (222 mg, 0.88 mmol, 1 equiv), BaCl_2_·2H_2_O (236 mg, 0.97 mmol) and dbqen (300 mg, 0.88 mmol, 1 equiv). Yield: 383 mg (82%, based on dbqen) of a green solid. Anal. Calcd (%) for C_22_H_28_Cl_2_N_4_O_4_V (*M*r = 534.33 g/mol): C, 49.41; H, 5.12; Cl, 13.26; N, 10.48; V, 9.53 Found (%): C, 49.61; H, 5.12; Cl, 13.23; N, 10.39, V, 9.22 (High resolution electrospray ionization mass spectrometry [HR-ESI(+)-MS]: calcd for *cis*-[V^IV^(O)(Cl)(dbqen)]Cl·3H_2_O (C_22_H_28_Cl_2_N_4_O_4_V) {[M-(Cl+3H_2_O)]^+^} *m*/*z* 444.0916, found 444.0904. μ_eff_ = 1.74 μ_B_

Crystals of *cis*-[V^IV^(O)(Cl)(dbqen)]ClO_4_·2CH_3_CN (**4’**) suitable for X-ray structure analysis were prepared as follows: Compound **4·**3H_2_O (50 mg, 0.09 mmol) was dissolved in water (10 mL) under magnetic stirring, and NaClO_4_ (11 mg, 0.09 mmol) was added to it. Upon addition of NaClO_4_, a light green precipitate was formed, which was filtered and dried. Dissolution of the green solid in CH_3_CN and layering of diethyl ether to it resulted in the formation of crystals of *cis*-[V^IV^(O)(Cl)(dbqen)]ClO_4_·2CH_3_CN.

#### *Cis*-fluorido[*N*,*N*′-Bis(8-quinolyl)cyclohexane-1,2-diamine-*N*,*N*,*N*,*N*]oxidovanadium(IV) Perchlorate, *cis*-[V^IV^(O)(F)(H_2_bqch)]ClO_4_ (**5**)

To a stirred solution of **1·**H_2_O (100 mg, 0.19 mmol) in water (20 mL) was added in one portion solid KF (12 mg, 0.21 mmol). Upon addition of KF, the light brown color of the solution changed to orange. The solution was stirred for an additional hour. Then solid NaClO_4_ (26 mg, 0.21 mmol) was added to it in one portion, and an orange precipitate was formed. The mixture was stirred for 3 h and filtered, washed with cold water (2 × 5 mL), and dried in vacuum to get 78 mg of the orange solid. Yield: 74% (based on **1·**H_2_O). Anal. Calcd (%) for C_24_H_24_ClFN_4_O_5_V (*M*_r_ = 553.63 g/mol): C, 52.02; H, 4.37; F, 3.43; N, 10.12; V, 9.20. Found (%): C, 52.01; H, 4.37; F, 3.40; N, 10.09; V, 9.19. (High resolution electrospray ionization mass spectrometry [HR-ESI(+-MS]: calcd for *cis*-[V^IV^(O)(F)(H_2_bqch)]ClO_4_· (C_24_H_24_ClFN_4_O_5_V) {[M-(ClO_4_)]^+^} *m*/*z* 454.1368, 454.1339 found; {[M-(F + H + ClO_4_)]^+^} *m*/*z* 434.1306, found 434.1285. μ_eff_ = 1.72 μ_B_

Crystals of **5·**CH_3_OH (**5’**) suitable for X-ray structure analysis were obtained by layering diethyl ether into a concentrated methyl alcohol solution of **5**.

#### *Cis*-fluorido[N,*N*′-Bis(8-quinolyl)ethane-1,2-diamine-*N*,*N*,*N*,*N*]oxidovanadium(IV) Perchlorate, *cis*-[V^IV^(O)(F)(H_2_bqen)]ClO_4_ (**6**)

Compound **6** was prepared using the same method reported for the synthesis of **5** with **3·**2H_2_O (93 mg, 0.19 mmol), KF (12 mg, 0.21 mmol), and NaClO_4_ (26 mg, 0.22 mmol) to get 78 mg of the orange solid. Yield: 74% (based on **3**·2H_2_O). Anal. Calcd (%) for C_20_H_18_ClFN_4_O_5_V (*M*r = 499.58 g/mol): C, 48.02; H, 3.63; F, 3.80; N, 11.21; V, 10.19 Found (%): C, 47.93; H, 3.60; F, 3.75; N, 10.96; V, 10.24 (High resolution electrospray ionization mass spectrometry [HR-ESI(+)-MS]: calcd for *cis*-[V^IV^(O)(F)(H_2_bqen)]ClO_4_**(**C_20_H_18_ClFN_4_O_5_V) {[M-(H+F+ClO_4_)]^+^} *m*/*z* 380.0837, found 380.0814. μ_eff_ = 1.74 μ_B_

Preparation of the compound *cis*-[V^IV^(O)(F)(H_2_bqen)]BF_4_ (**6’**) was performed with the same method as for the synthesis of **6** except that NH_4_BF_4_ was used instead of NaClO_4_. Crystals of **6’**suitable for X-ray structure analysis were obtained by dissolving **6’** into methyl alcohol and layering diethyl ether into the concentrated methyl alcohol solution of *cis*-[V^IV^(O)(F)(H_2_bqen)]BF_4_.

## Results and Discussion

### Synthesis of the Ligands and Oxidovanadium(IV) Compounds

**Scheme 3:**
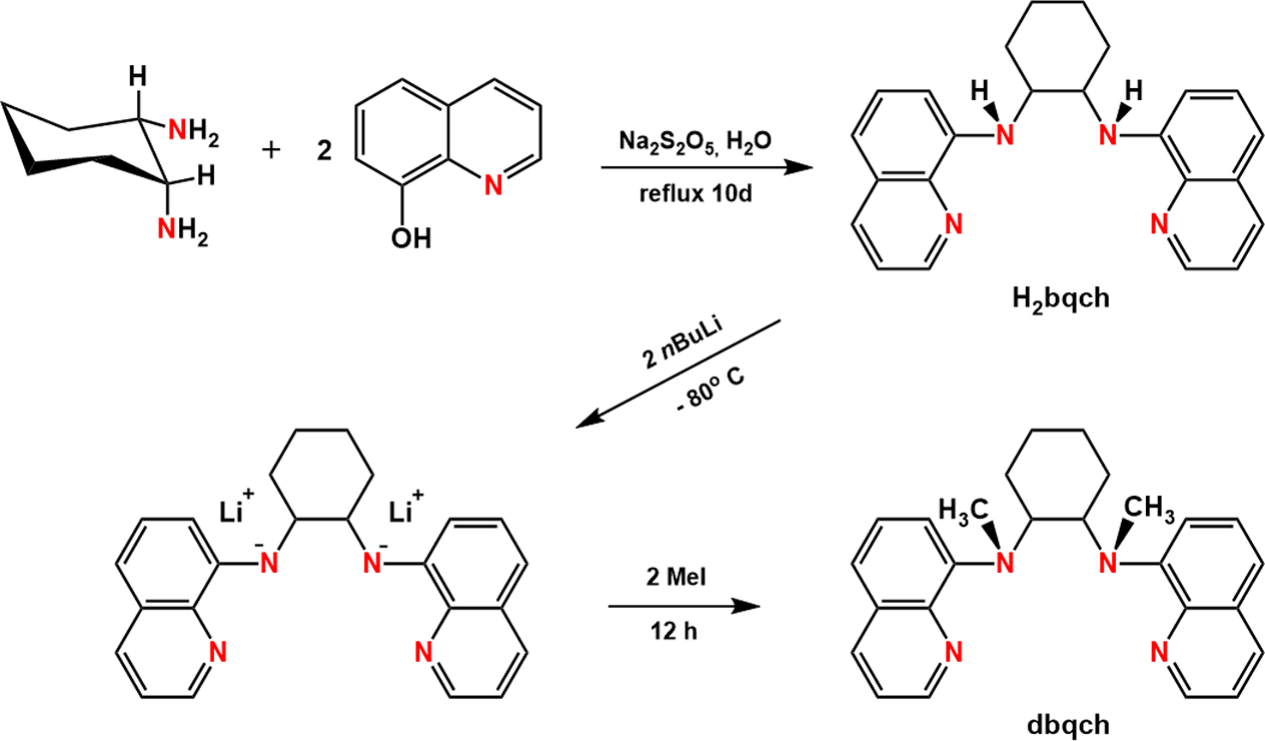
Synthesis of the Ligands H_2_bqch and dbqch

The synthesis of the ligands dbqch and dbqen is depicted in Scheme 3 and includes three steps: The first step involves the reaction of the diamine (1 equiv) with 8-hydroxyquinoline (2 equiv) to get the secondary amines H_2_bqch and H_2_bqen. The secondary amines were prepared by slight modification of the method of Britovsek^46^ and co-workers to increase their yield by 10–15%. The second step involves the deprotonation of the secondary amines with 2 equiv of *n*-BuLi. In the third step, the methylation by CH_3_I (two equivalents) of the deprotonated amines was carried out to afford the dimethylated ligands.

**Scheme 4:**
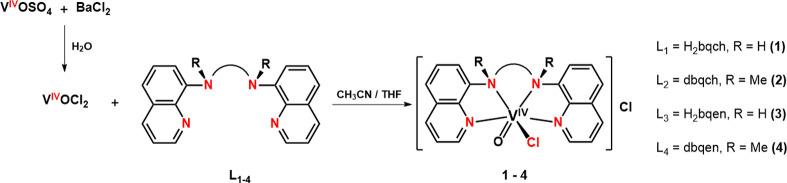
Synthesis of the *cis*-[V^IV^(O)(Cl)(N_4_)_nm/m_]^+^ Compounds (**1**–**4**)

**Scheme 5:**
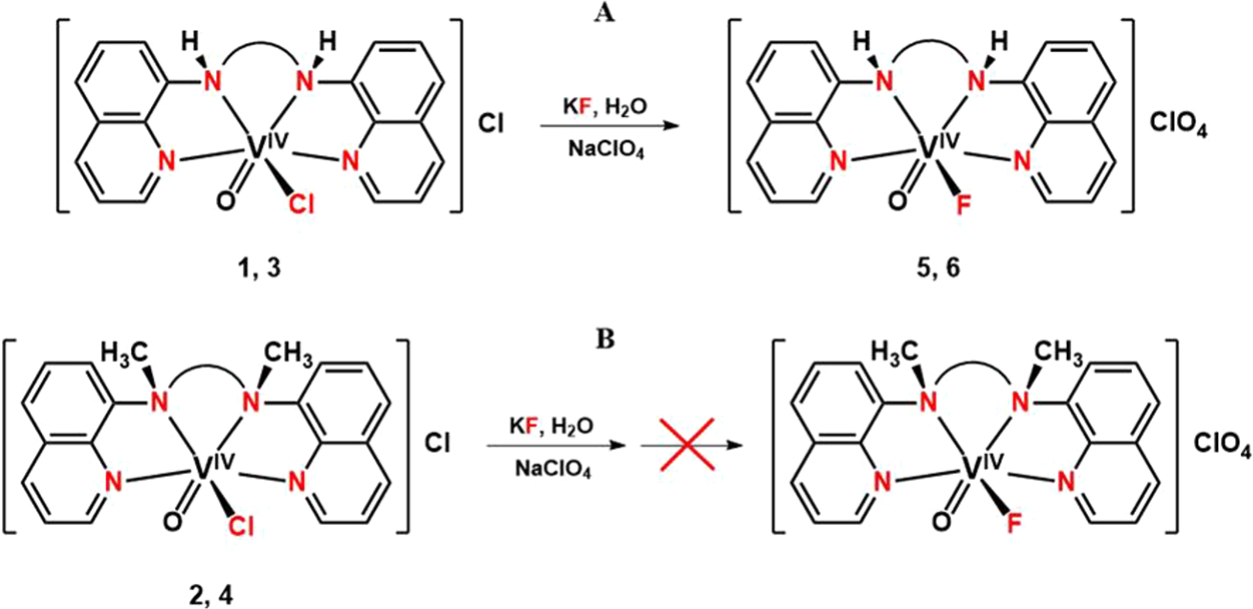
Synthesis of *cis*-[V^IV^(O)(F)(H_2_bqch)]^+^ (**5**) and *cis*-[V^IV^(O)(F)(H_2_bqen)]^+^ (**6**) (A). The methylated *cis*-[V^IV^(O)(Cl)(N_4_)_m_]^+^ derivatives do not react with F^–^ (B)

The synthesis of the *cis*-[V^IV^(O)(Cl)(N_4_)_nm/m_]^+^ and *cis*-[V^IV^(O)(F)(N_4_)_nm_]^+^ compounds is shown in Schemes 4 and 5. respectively. (The indexes nm and m mean the nonmethylated and methylated ligands respectively.) The *cis*-[V^IV^(O)(F)(N_4_)_m_]^+^ derivatives were not synthesized, since the species *cis*-[V^IV^(O)(Cl)(N_4_)_m_]^+^ does not react with F^–^ due to steric hindrance (see EPR and DFT calculations for details).

### Crystal Structures

Crystallographic data and selective bonds and angles, for complexes **2’**, **2”**, **4’**, **5’,** and **6’**, are summarized in Tables 1, 2, S1 and S2.

**Table 1:** Crystal Data and Details of the Structure Determination for the V^IV^O^2+^ Compounds

parameter	[VOCl(dbqch)]ClO_4_·2CH_3_CN	[VOCl(dbqen)]ClO_4_·2CH_3_CN	[VOF(H_2_bqch)]ClO_4_·CH_3_OH	[VOF(H_2_bqen)]BF_4_
empirical formula	C_30_H_34_Cl_2_N_6_O_5.16_V	C_26_H_28_Cl_2_N_6_O_5_V	C_25_H_24_FClN_4_O_6_V	C_20_H_18_F_5_BN_4_OV
formula weight	682.96	626.38	581.87	487.13
temperature	100(2) K	150 K	150 K	150 K
wavelength	0.71073	0.71073	0.71073	0.71073
space group	*P* 21/*c*	*P*-1	*P* 21/*c*	*Fdd*2
*a*(Å)	17.1586(14)	7.4869(5)	16.2714(10)	31.268(8)
*b*(Å)	12.2319(5)	12.6366(8)	15.9079(10)	9.975(3)
*c*(Å)	16.3466(14)	16.0650(11)	10.4324(6)	12.717(3)
α (deg)	90	106.640(3)	90	90
β (deg)	118.116(11)	97.314(4)	95.596(3)	90
γ (deg)	90	99.829(3)	90	90
vol. (Å^3^)	3026.0(5)	1409.66(16)	2687.5(3)	3966.7(17)
*Z*	4	2	4	8
ρ (g/cm^–3^)	1.499	1.476	1.438	1.631
Abscoeff (mm^–1^)	0.566	0.589	0.522	0.568
R1^a^	0.0950	0.0371	0.0435	0.0425
wR2^b^	0.2305	0.1014	0.1231	0.0893
GoF, S^c^	1.040	1.045	1.041	1.124
R-Factor (%)	9.50	3.71	4.35	4.25

a R1 = Σ||*F*_o_| – |*F*_c_||/Σ|*F*_o_|.

b *w* R2 = {Σ[*w*(*F*_o_^2^ – *F*_c_^2^)^2^]/Σ[*w*(*F*_o_^2^)^2^]}^1/2^, where *w* = 1/[σ^2^(*F*_o_^2^) + (*aP*)^2^ + *bP*], *P* = (*F*_o_^2^ + 2*F*_c_^2^)/3.

c GoF = {Σ[*w*(*F*_o_^2^ – *F*_c_^2^)^2^]/(*n* – *p*)}^1/2^, where *n* = number of reflections and *p* is the total number of parameters refined.

**Table 2:** Interatomic Distances (Å) and Angles (deg) Relevant to the V^IV^ Coordination Sphere

parameter	[VOCl(dbqch)]ClO_4_·2CH_3_CN	[VOCl(dbqen)]ClO_4_·2CH_3_CN	[VOF(H_2_bqch)]ClO_4_·CH_3_OH	[VOF(H_2_bqen)]BF_4_
V(1) - X^a^	2.178(2)	2.3265(7)	1.834(1)	1.730(1)^b^
V(1) - N(1)	2.102(4)	2.098(1)	2.132(2)	2.107(2)
V(1) - N(2)	2.337(6)	2.366(2)	2.291(2)	2.245(2)
V(1) - N(3)	2.184(4)	2.198(2)	2.173(2)	2.245(2)
V(1) - N(4)	2.104(4)	2.094(1)	2.104(2)	2.107(2)
V(1) - O(1)^a^	1.643(4)	1.609(1)	1.626(2)	1.626(2)
X - V(1) - N(1)	91.70(1)	88.76(5)	88.36(7)	98.08(7)
X - V(1) - N(2)	88.76(1)	89.02(4)	84.82(7)	88.13(7)
X - V(1) - N(3)	163.22(1)	165.55(5)	155.46(7)	161.70(7)
X - V(1) - N(4)	92.34(1)	94.19(5)	89.50(7)	91.70(7)
X - V(1) - O(1)	102.61(1)	102.30(5)	107.25(7)	107.92(7)
N(1) - V(1) - N(2)	74.37(2)	75.81(6)	73.73(7)	77.02(7)
N(1) - V(1) - N(3)	95.62(2)	93.44(6)	102.48(7)	89.96(7)
N(1) - V(1) - N(4)	169.33(2)	164.54(6)	171.93(8)	163.37(8)
N(2) - V(1) - N(3)	78.75(2)	77.70(6)	77.40(7)	77.64(7)
N(2) - V(1) - N(4)	95.85(2)	89.04(6)	98.33(7)	89.96(7)
N(3) - V(1) - N(4)	78.00(2)	80.05(6)	76.68(7)	77.02(7)
O(1) - V(1) - N(1)	92.85(2)	95.38(7)	91.87(8)	91.70(8)
O(1) - V(1) - N(2)	163.28(2)	165.62(7)	161.19(8)	161.70(8)
O(1) - V(1) - N(3)	92.11(2)	91.73(7)	94.48(8)	88.13(8)
O(1) - V(1) - N(4)	95.89(2)	98.81(7)	96.19(8)	98.08(8)

a X corresponds to Cl(1) for [VOCl(dbqch)]ClO_4_·2CH_3_CN and [VOCl(dbqen)]ClO_4_·2CH_3_CN. X corresponds to F(1) for [VOF(H_2_bqch)]ClO_4_·CH_3_OH and to F(1) or O(1) for [VOF(H_2_bqen)]BF_4_.

b In this structure, there is a disorder between oxygen and fluorine atoms, and thus, the reported *d*(V–F) in Table 2 is a mean value of the *d*(V–F) and *d*(V = O).

**Figure 1:**
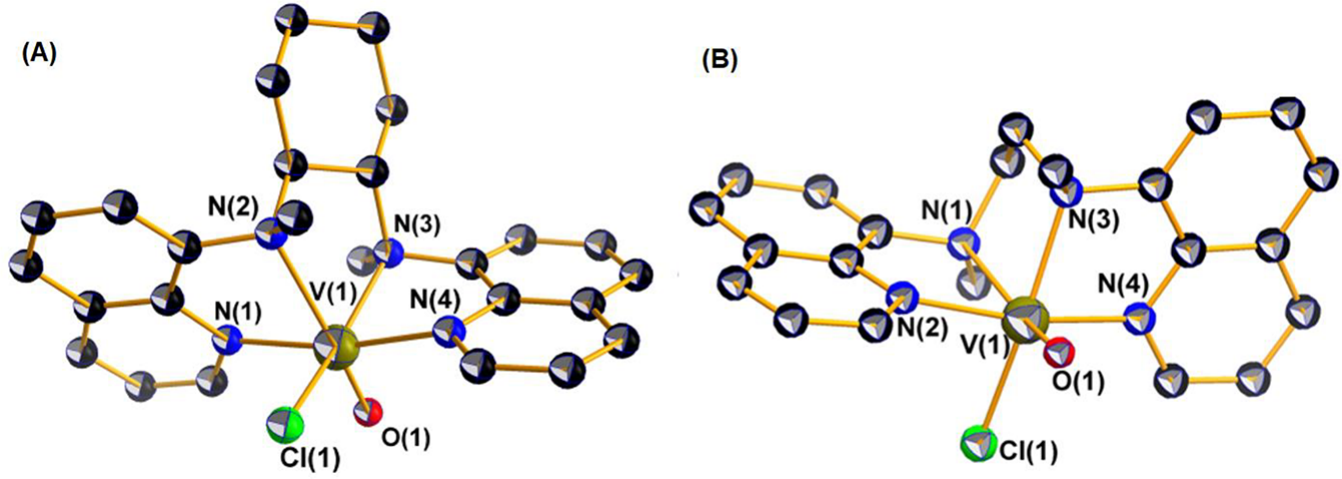
ORTEP plot of the cations of **2’** (A) and **4’** (B) (the dimethylated-chlorido derivatives), with 50% thermal ellipsoids. Hydrogen atoms are omitted for clarity.

A perspective view of the structure of the cation of **2”**, *cis*-[V^IV^(O)(Cl)(dbqch)]^+^ with the atomic numbering scheme used is shown in Figure 1A. The structure of [V^IV^(O)(Cl)(dbqch)]^+^ reveals that the ligand adopts a *cis*-α topology around the vanadium(IV) center with the two quinoline rings trans to each other and the two N–CH_3_ groups in an anticonformation. The vanadium(IV) atom in [V^IV^(O)(Cl)(dbqch)]^+^ is bonded to a tetradentate (*N*_q_*,N*_a_*,N*_a_*,N*_q_) dbqch ligand, and an oxido and chlorido ligands. The donor atoms surrounding the vanadium(IV) atom are disposed in a severely distorted octahedral geometry where the two quinoline nitrogens N(1) and N(4), the amine nitrogen atom N(3) and the chloride ion occupy the equatorial plane, while the amine nitrogen atom N(2) and the oxido ligand occupy the axial positions. The dbqch ligand forms three five-membered fused chelate rings. The vanadium(IV) bond distances of the *trans*-quinoline nitrogens [V^IV^–N(1) = 2.102(4) and V^IV^–N(4) = 2.104(4) Å] are noticeably shorter than those of the amine nitrogens N(2) and N(3) [2.337(4) and 2.184(4) Å]. The two V^IV^–N_amine_ bond lengths are substantially different due to the strong trans influence of the oxido ligand. The long V(1)–N(2) bond length [2.337(4) Å] shifts the equatorial N(3) amine donor atom 0.198 (5) Å for **2”** [0.245 (3) Å for **2’**] under the equatorial plane defined by the two quinoline N and the Cl donor atoms. Consequently, the quinoline ring, parallel to equatorial plane, tilts, forming a 17.7(1) ^o^ for **2”** [16.3(1) ^o^ for **2’**] angle with the equatorial plane.

The *d*(V^IV^=O) of 1.643(4) Å lies in the upper limit of the range observed for oxidovanadium(IV) complexes (1.56–1.66 Å),^48−52^ while the *d*(V^IV^–Cl) of 2.178(2) Å lies in the expected range.^50,52,53^ The structure of **4’** (Figure 1B) has very similar structural features with those of **2’**: therefore, it will not be discussed.

**Figure 2:**
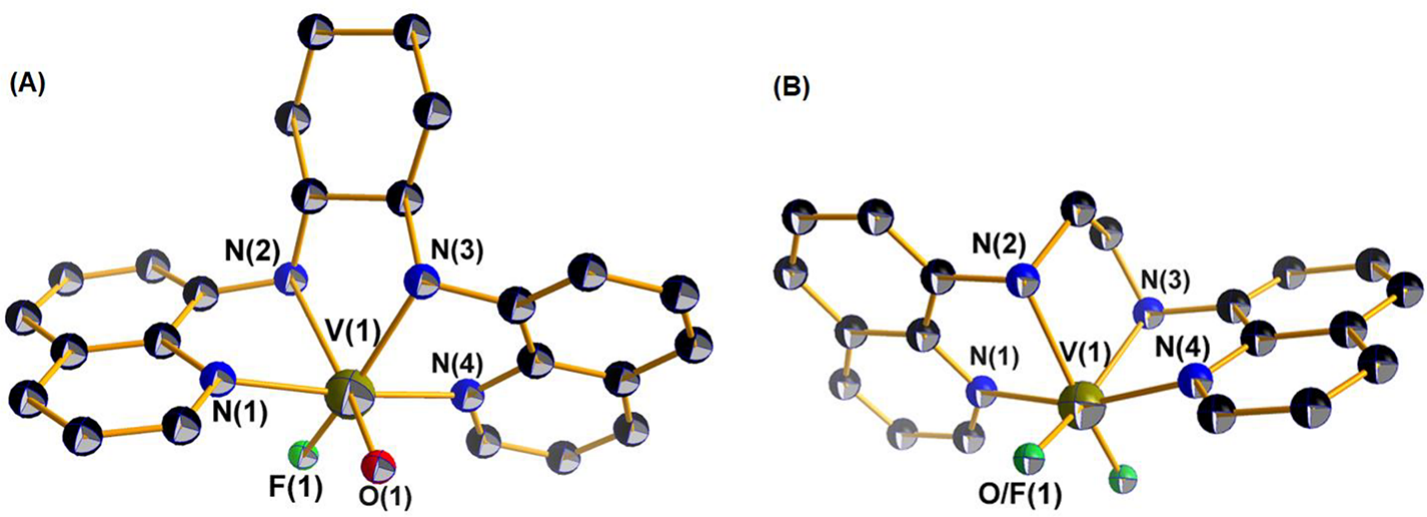
ORTEP plot of the cations of **5’** (A) and **6’** (B) (the amine-fluorido derivatives), with 50% thermal ellipsoids. Hydrogen atoms are omitted for clarity.

The molecular structures of the *cis*-[V^IV^(O)(F)(H_2_bqch)]ClO_4_·CH_3_OH (**5’**) and *cis*-[V^IV^(O)(F)(H_2_bqen)]BF_4_ (**6’**) are depicted in Figure 2A and 2B, respectively. In *cis*-[V^IV^(O)(F)(H_2_bqch)]ClO_4_·CH_3_OH (**5’**), the vanadium(IV) atom is coordinated to two quinoline N atoms, two secondary amine N atoms, a fluorine atom, and an oxygen atom. The vanadium adopts a highly distorted octahedral geometry and is displaced above the mean equatorial plane, defined by the two quinoline N atoms, one secondary amine N, and a fluorine atom, by 0.256 Å toward the oxido ligand. The long V(1)–N(2) bond length [2.292(2) Å] shifts the equatorial N(3) amine donor atom 0.465 (2) Å under the equatorial plane defined by the two quinoline N and the F donor atoms (Scheme 2). The quinoline ring, parallel to the equatorial plane, tilts, forming a 16.50(6)° angle with the equatorial plane. The fluorine atom is coordinated in a cis position to the oxido ligand and the *d*(V^IV^–F) of 1.834(1) Å has been, to our knowledge, the shortest observed for oxyfluoride vanadium(IV) compounds.^54−57^ Compound *cis*-[V^IV^(O)(F)(H_2_bqen)]BF_4_ (**6’**) has similar structural characteristics: therefore, it will not be discussed.

### IR Spectroscopy

The IR spectra of the six oxidovanadium(IV) compounds exhibit a very strong and sharp band in the range 962–977 cm^–1^, which was assigned to ν[V^IV^(O)] (see Figures S1–S6). The IR spectra of **5** and **6** (the fluorido compounds) reveal a moderate sharp band at 563 and 557 cm^–1^ respectively (see Figures S5–S6), which is missing in the spectra of the chlorido compounds **1**–**4** (see Figures S1–S4), and this band was assigned to ν(V^IV^–F).

### Catalytic Evaluation

The present oxidovanadium(IV) compounds **1**–**6** were used as catalysts for the cyclohexane oxidation with H_2_O_2_ (30% w/w) at room temperature (25 ± 0.5 °C). In catalytic reactions, the used molar ratio was [catalyst: H_2_O_2_: cyclohexane] = [1:1000:2500 μmoles] in the presence or not of 100 μmoles of HCl. Catalytic data including product yields (%), TON, and TOF are given in Table 3.

**Table 3:** Catalytic Oxidation of Cyclohexane by Oxidovanadium(IV) Complexes **1**–**6** in the Presence of H_2_O_2_

V^IV^O-compounds	products	yield (%)^c^	TON^d^	TOF (h^–1^)^e^
**1**^a^	cyclohexanol	17.7	241	40.2
	cyclohexanone	6.4		
**1**^b^	cyclohexanol	15.2	203	33.8
	cyclohexanone	5.1		
**2**^a^	cyclohexanol	5.0	77	12.8
	cyclohexanone	2.7		
**2**^b^	cyclohexanol	14.3	195	32.5
	cyclohexanone	5.2		
**3**^a^	cyclohexanol	15.2	213	35.5
	cyclohexanone	6.1		
**3**^b^	cyclohexanol	13.2	198	33
	cyclohexanone	6.6		
**4**^a^	cyclohexanol	6.0	102	17
	cyclohexanone	4.2		
**4**^b^	cyclohexanol	10.8	173	28.8
	cyclohexanone	6.5		
**5**^a^	cyclohexanol	9.5	111	18.5
	cyclohexanone	1.6		
**5**^b^	cyclohexanol	21.6	241	40.2
	cyclohexanone	2.5		
**6**^a^	cyclohexanol	18.6	293	48.8
	cyclohexanone	10.7		
**6**^b^	cyclohexanol	15.7	234	39
	cyclohexanone	7.7		

a Conditions: molar ratio of [catalyst:H_2_O_2_:substrate]= [1:1000:2500] in 1 mL of CH_3_CN (1 equiv = 1 μmol).

b Conditions: molar ratio of [catalyst:HCl:H_2_O_2_:substrate]= [1:100:1000:2500] in 1 mL of CH_3_CN (1 equiv = 1 μmol).

c Yields based on the starting substrate and products formed. The mass balance is 98–100%. The reaction time was 6 h.

d TON: total turnover number, moles of products formed per mole of catalyst.

e TOF: turnover frequency which is calculated by the expression [products]/[catalyst] × time (h^–1^).

**Figure 3:**
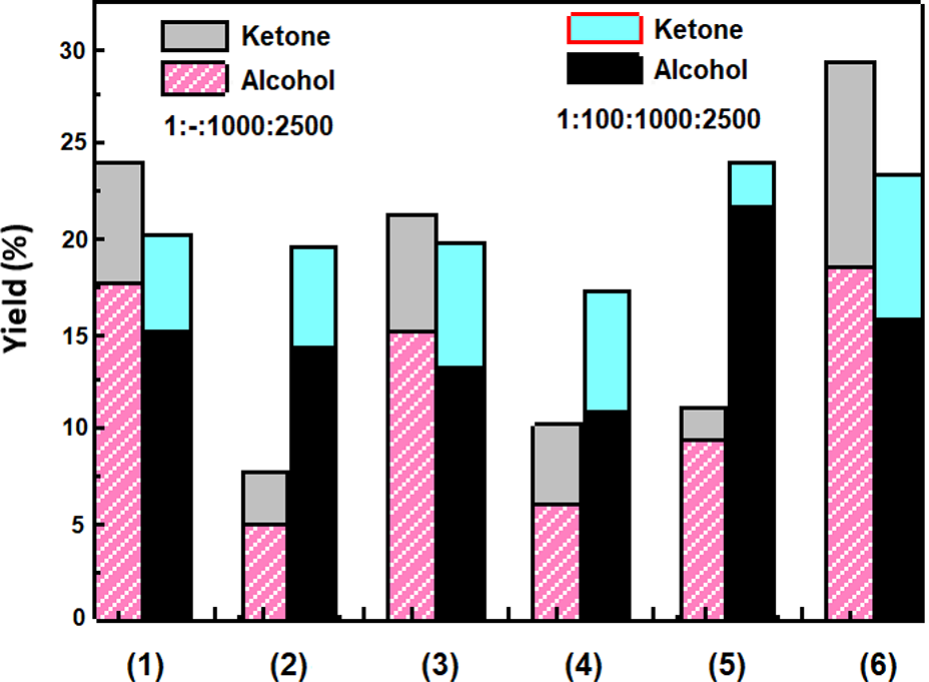
Distribution of oxidation products catalyzed by the oxidovanadium(IV) compounds **1**–**6** in the presence of H_2_O_2_. Conditions: molar ratio of [catalyst:H_2_O_2_:substrate]= [1:1000:2500] in 1 mL CH_3_CN (1 equiv = 1 μmol) or molar ratio of [catalyst:HCl:H_2_O_2_:substrate] = [1:100:1000:2500] in 1 mL CH_3_CN (1 equiv = 1 μmol). Yields based on the starting substrate and products formed. The reaction time was 6 h. See Table 3 for further details on reaction conditions.

**Figure 4:**
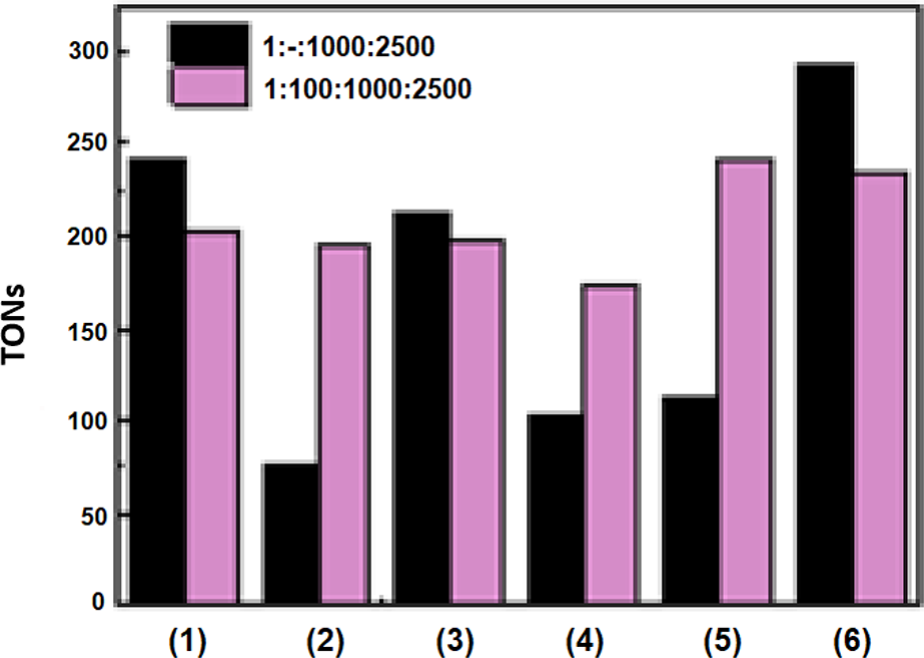
Turnover frequency of the oxidation of cyclohexane catalyzed oxidovanadium(IV) compounds **1**–**6**. Conditions: molar ratio of [catalyst:H_2_O_2_:substrate] = [1:1000:2500] in 1 mL CH_3_CN (1 equiv = 1 μmol) or molar ratio of [catalyst:HCl:H_2_O_2_:substrate] = [1:100:1000:2500] in 1 mL of CH_3_CN (1 equiv = 1 μmol). Yields based on the starting substrate and products formed. The reaction time was 6 h. See Table 3 for further details on reaction conditions.

According to Table 3, the oxidovanadium(IV) compounds **1**–**6** are able to oxidize cyclohexane with hydrogen peroxide at room temperature. More specifically, oxidation of cyclohexane catalyzed by **1** and **3** produced cyclohexanol and cyclohexanone with 17.7, 6.4% and 15.2, 6.1% yields, respectively resulting in 24.1 and 21.3% total yields (Figure 3). The addition of 100 μmoles of HCl in the catalytic reaction led to reduced total yields, i.e., 20.3 and 19.8% for **1** and **3**, respectively. The corresponding methylated oxidovanadium(IV) compounds **2** and **4** without HCl provided total yields for cyclohexane oxidation 7.7 and 10.2%, respectively, which increased in the presence of 100 μmoles of HCl, 19.5% and 17.3%, respectively (Figure 3). The nonmethylated fluorido compounds **5** and **6** gave total yields 11.1% and 29.3%, respectively, which were higher than their chlorido analogues (Table 3). Cyclohexane oxidation catalyzed by **5** and **6** was not affected by HCl as a promoter; its presence resulted in even lower yields (24.1% and 23.4% respectively). TONs achieved by the catalysts **1**–**6** ranged from 77 to 293 and are visualized in Figure 4.

Based on our catalytic data, the addition of HCl to the cyclohexane oxidation, catalyzed by the methylated oxidovanadium(IV) compounds **2** and **4**, increases the yield of oxidation products. For the nonmethylated compounds **1**, **3**, **5**, and **6**, the addition of HCl decreases the catalytic activity. Analogous negative effect on catalytic cyclohexane oxidation was observed when HCl was replaced by 2-pyrazine carboxylic acid (PCA) or HNO_3_ (data not shown). The use of PCA as promoter in alkane oxidation catalyzed by oxidovanadium(IV) compounds is well-known due to its assistance for H^+^ migration from a coordinated H_2_O_2_ to the oxido-ligand.^45^ Here, the observed chemical behavior of **1**, **3**, **5**, and **6** reveals that the two −NH– groups in conjunction with the oxido-ligand are able to manage the hydrogen peroxide deprotonation which is coordinated to vanadium center toward homolytic O–O bond cleavage and generation of ^•^OH radicals. Cyclohexane oxidation most probably occurs via these ^•^OH radicals which abstract a cyclohexane hydrogen atom to form cyclohexyl radicals. The alkyl radicals in oxygenated organic solvents readily form alkyl hydroperoxides (cyclohexyl hydroperoxide in our case) as primary intermediate oxidation product which transformed to cyclohexanol and cyclohexanone.^58−60^

### Magnetism and X-Band Continuous-Wave (cw) EPR Spectra of **1**–**6**

The magnetic moments of compounds **1**–**6**, at 298 K, have magnetic moments in the range of 1.70–1.74 μ_B_, in accord with the spin-only value expected for *d*^1^, *S* = 1/2 systems. These μ_eff_ values constitute clear evidence that the oxidation of vanadium in **1**–**6** is IV.

**Figure 5:**
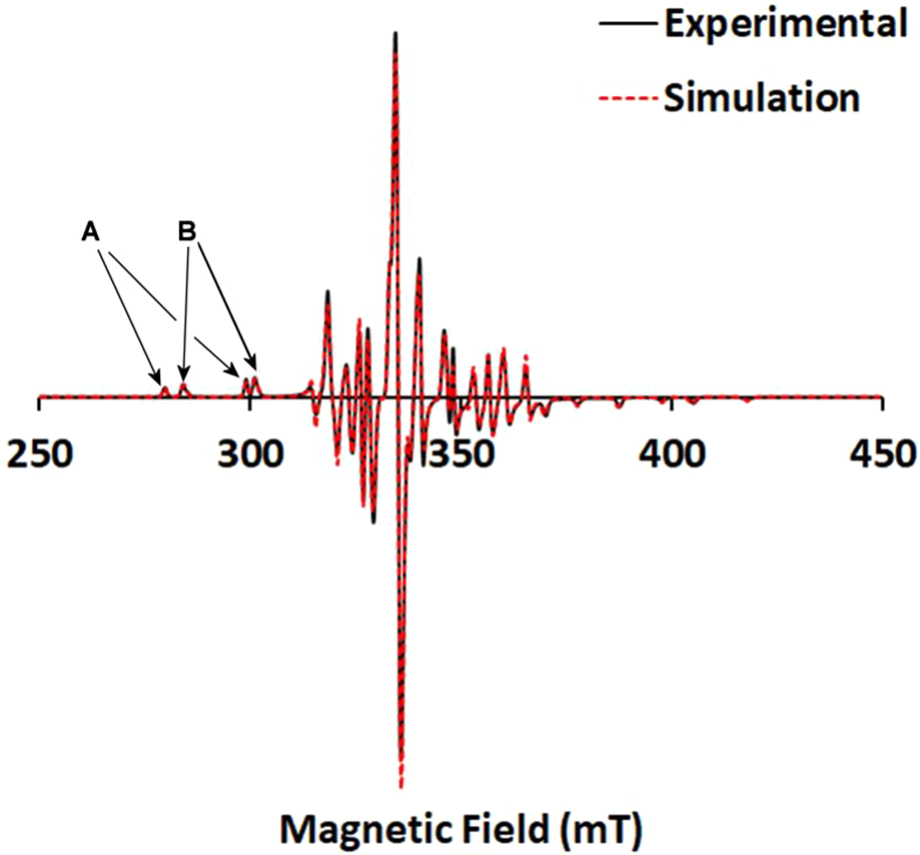
X-band cw EPR spectrum of a frozen solution of the compound *cis*-[V^IV^(O)(Cl)(dmbqch)]Cl (**2**, species **A** and **B**) in DMSO (1.00 mM) at 120 K and its simulated spectrum.

**Scheme 6:**
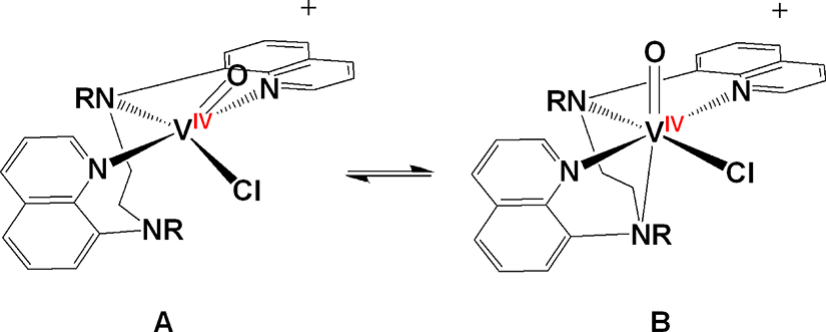
Equilibrium of the Five- (**A**) *cis*-[V^IV^(O)(Cl)N_4_]^+^ Oxidovanadium(IV) Compounds (**1**–**6**) in Solution (DMSO) and Six-Coordinate (**B**) The structure **B** is identical with that determined from the single-crystal X-ray structure analysis of **2** and **4**.

The X-band cw EPR parameters, of the frozen (120 K) solutions (DMSO) of the oxidovanadium(IV) compounds **1**–**6** are depicted in Table 4 and were calculated from the simulation of their experimental EPR spectra. The spectra of the compounds *cis*-[V^IV^(O)(Cl)(H_2_bqen)]Cl·2H_2_O (**3·**2H_2_O), *cis*-[V^IV^(O)(F)(H_2_bqch)]ClO_4_ (**5**) (Figure S7), and *cis*-[V^IV^(O)(F)(H_2_bqen)]ClO_4_ (**6**) (Figure S8) were successfully simulated assuming one V^IV^ species in solution, while the spectra of *cis*-[V^IV^(O)(Cl)(H_2_bqch)]Cl·H_2_O (**1·**H_2_O), *cis*-[V^IV^(O)(Cl)(dmbqch)]Cl (**2**) (Figure 5), and *cis*-[V^IV^(O)(Cl)(dmbqen)]Cl·3H_2_O (**4·**3H_2_O) (Figure S8) were successfully simulated assuming the coexistence of another species (B) in equilibrium with A (Scheme 6). The spectra of **1**–**4** were simulated considering axial symmetry, while those of **5** and **6** were simulated with respect to rhombic symmetry. For the simulations of the spectra of **5** and **6**, the superhyperfine coupling of the electron spin with the neighboring F^–^ was also included in the Hamiltonian. The *A*_||_ values of **1**–**4** are ∼177 × 10^–4^ and ∼155 × 10^–4^ cm^–1^ for the species A and B, respectively, and the *A*_*z*_ values of **5** and **6** are ∼179 × 10^–4^ cm^–1^.

**Table 4:** Cw X-Band EPR Parameters of the DMSO Frozen Solutions of Oxidovanadium(IV) Compounds **1**–**6**^a^

compound	*g*_*x*_,*g*_*y*_(*g*_⊥_)	*g*_*z*_(*g*_||_)	*A*_*x*_,*A*_*y*_(*A*_⊥_) × 10^–4^ (cm^–1^)	*A*_*z*_(*A*_||_) × 10^–4^ (cm^–1^)	*A*_x(F)_,*A*_y(F)_(*A*_⊥(F)_)× 10^–4^ (cm^–1^)	*A*_z(F)_(*A*_||(F)_) × 10^–4^ (cm^–1^)	equatorial coordination environment
**1** (A, 79%)	1.971	1.929	–65.9	–177.6			N_3_Cl
**1** (B)	1.976	1.947	–54.9	–155.6			N_2_Cl
**2** (A, 35%)	1.970	1.928	–65.7	–177.5			N_3_Cl
**2** (B)	1.974	1.952	–53.4	–156.9			N_2_Cl
**3** (A)	1.970	1.928	–65.8	–177.4			N_3_Cl
**4** (A, 24%)	1.971	1.928	–66.0	–177.4			N_3_Cl
**4** (B)	1.975	1.953	–53.8	–157.2			N_2_Cl
**5** (A)	1.976, 1.965	1.927	–65.5, −65.6	–178.8	15.5, 20.8	10.4	N_3_F
**6** (A)	1.976, 1.964	1.928	–65.6, −65.6	–179.2	13.5, 18.2	9.7	N_3_F

a The A and B correspond to the two identified isomers depicted in Scheme 6.

**Scheme 7:**
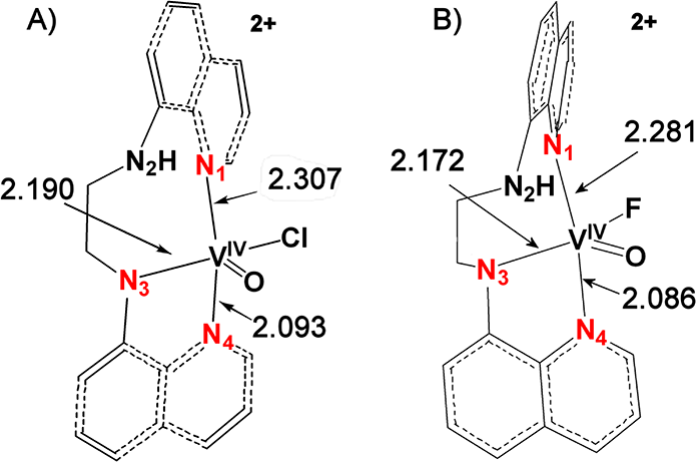
Equilibrium Geometry of the (A) [V^IV^(=O)(Cl)(N_4_H)]^2+^, τ = 0.51, *d*(V···N_2_) = 3.303 Å) and (B) [V^IV^(=O)(F)(N_4_H)]^2+^, τ = 0.70, *d*(V···N_2_)= 3.341 Å, in Acetonitrile Solution Optimized at the PBE0/Def2-TZVP(V)^υ^6-31+G(d)(E)/PCM Level of Theory and Selected Bond Distances in Å

Theoretical calculations support the presence of two possible minimum energy structures for the compounds **1**–**6** in solution, (a) the six-coordinate distorted octahedral structure (Scheme 6B) found in the single crystal structures of **2**, **4**, **5**, and **6** and (b) a five-coordinate species (Scheme 6A) formed from the dissociation of the axial to oxido group amine nitrogen atom, exhibiting a highly distorted trigonal bipyramidal structure (Scheme 7) with a trigonality index τ = 0.51 [τ = (a–b)/60 = 0.51; a = N(3)–V–Cl = 134.0°, b = N(1)–V–N(4) = 164.7°).^61^ Other possible structures of **1**–**6** in solution, such as five-coordinate species formed from the dissociation of Cl^–^, result in high energy species based on theoretical calculations (vide infra). In addition, conductivity measurements of the DMSO and CH_3_CN solutions of **1** – **6** gave values 55–65 cm^–1^ mol^–1^ Ω^–1^ (DMSO) and 130–150 cm^–1^ mol^–1^ Ω^–1^ (CH_3_CN), as expected for 1:1 electrolytes. Thus, it is clear from the conductivity measurements that the Cl^–^ or F^–^ donor atoms do not dissociate in solution. DFT calculations of the EPR parameters of **1** (structure A) and **1** (structure B) at the BHandHLYP/6-311g (d,p) level of theory predict *Az* = −173.8 × 10^–4^, *A*_*y*_ = −75.2 × 10^–4^, *A*_*x*_ = −71.6 × 10^–4^ and *Az* = −152.8 × 10^–4^, *A*_*y*_ = −56.9 × 10^–4^, *A*_*x*_ = −54.1 × 10^–4^ for the five-coordinate (structure A) and six-coordinate (structure B) respectively. The theoretically predicted *A*_*z*_ values are ∼2.5% lower than the experimental, due to the accuracy of the method used, and this deviation is similar to the deviation reported for the Gaussian calculations of charged vanadium complexes at the same level of theory.^62^

The *A*_||_ or *A*_*z*_ parameters depend on the donor atoms in the equatorial plane of the vanadium(IV) compounds and can be calculated from the empirical additivity relationship (eq 4).^63,64^
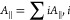
4*A*_*||,i*_ is the contribution of each donor atom to *A*_||_.

The donor atoms in the equatorial plane of **1**–**4** consist of a Cl^–^, two quinoline N (N_q_), and one aromatic amine N (N_ArNH2_) atoms. The *A*_*||,i*_ contributions N_q_ and N_ArNH2_ have not been determined previously. The *A*_*||,i*_ value of other aromatic heterocyclic N donor atoms, such as imidazole, pyridine, etc., and N_RNH2_ were used instead for the contribution of N_q_ and N_ArNH2_ respectively.^65^ The calculated *A*_||_ value using eq 4 is approximately −165 × 10^–4^ cm^–1^. However, the experimental and the calculated values of *A*_||_ are significantly lower for the octahedral species B and significantly higher than the five-coordinate species A.

The dramatic decrease of the experimental *A*_||_ values of species B, compared with the values calculated from the additivity relationship, is attributed to the coordination of the amine nitrogen in the axial position trans to the oxido group (Scheme 6).^66^ Tolis et al. have also suggested that axial donor atoms induce a radial expansion of the vanadium *d*_*xy*_ orbital, resulting in a reduced electron density on the V^IV^ and decrease of *A*_*z*_.^67^

On the other hand, the much higher *A*_*z*_ (−177.5 × 10^–4^ cm^–1^) experimental values of species A in comparison to the predicted *A*_*z*_ values for **1**–**6** from the additivity relationship are attributed to the distortion in the equatorial plane by the elongation of V–N(1) (2.307 Å), due to the tension in the N(3)---N(1) eight-membered ring (Scheme 7). Apparently, the weakening of the bonding at the equatorial plane results in an increase of *A*_*z*_ values.

**Scheme 8:**
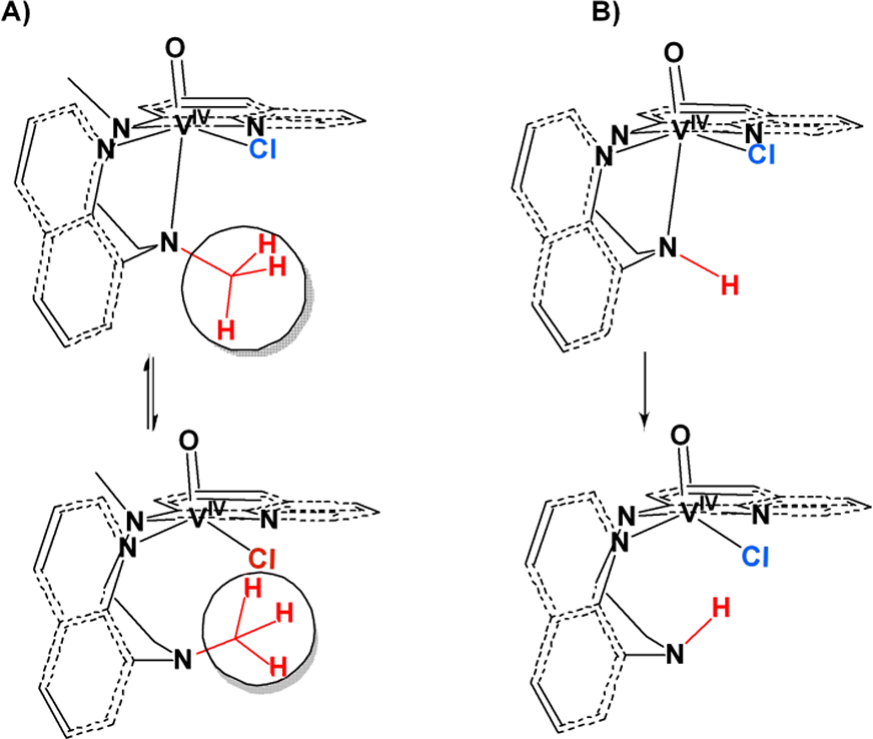
(A) Possible Mechanism with Which the Steric Hindrance of the Bulky Methyl Group Forces the V^IV^ Compounds to Acquire the Six-Coordinate Structure in Solution. (B) In the Absence of Steric Hindrance, the Compounds Adopt the Five-Coordinate Structure in Solution

The equilibrium between species **A** and **B** is shifted toward **B**, when the amine hydrogen atoms of the ligands (H_2_bqch, H_2_bqen, in compounds **1**, **3**) are replaced with the bulky methyl groups (dbqch, dbqen in compounds **2**, **4**). In addition, theoretical calculations revealed that **3**(**A**) is thermodynamically more stable than **3**(**B**), whereas **4**(**B**) is thermodynamically more stable than **4**(**A**). In addition, from the quantities of **B** in the solution being 21% and 0% for the compounds **1** (the cyclohexane derivative) and **3** (the ethylenediamine derivative), respectively, it is reasonable to conclude that cyclohexane-1,2-diamine chelate ring is more rigid than the 1,2-ethylenediamine one. The chelate ring defined by the vanadium(IV) atom and the two amine nitrogen atoms is stretched due to the elongation of the bond V^IV^–N_am.axial_. Moreover, the attachment of the methyl groups to the amine nitrogen atoms increases the steric interactions between Cl^–^ and the −CH_3_ group (Scheme 8) forcing equatorial N_amine_ to remain ligated to vanadium nucleus, forming the six-coordinate species B (Scheme 6). Dissociation of the equatorial N_amine_ atom results in the formation of an eight-membered chelate ring (Scheme 7) similar to the chelate rings for other V^IV^ compounds reported and characterized by crystallography.^68^

The EPR parameters calculated from the simulation of the experimental spectra reveal that compounds **5** and **6** acquire the structure A in DMSO. This might be attributed to the stronger trans effect of F^–^ than Cl^–^ on N(3) (Scheme 7). On the basis of the additivity relationship,^63,64,69,70^ and considering *A*_*z*_ contribution for F^–^, either −40.1 × 10^–4^ cm^–1^ {*cis*-[V^IV^(O)(F)(4,4′-dtbipy)_2_]BF_4_}^55^ or −41.8 × 10^–4^ cm^–1^ {[V^IV^(O)(F)_2_(DMSO)_3_]},^71^ one would expect lower experimental *A*_*||*_ values for **5** and **6** in comparison to those of **1**–**4** (Table 4). In marked contrast, the *A*_*z*_ values of **5** and **6** were slightly higher. Theoretical calculation of **6**(B) (Scheme 7) at BHandHLYP/6-311g (d,p) level gives a value for *A*_*z*_ (−178.3 × 10^–4^ cm^–1^) which is very close to the experimental one. The higher experimental *A*_*z*_ values of **5** and **6** than **1**–**4** and the higher *A*_*z*_ calculated values using eq 4, are attributed to higher trigonality index of **5** and **6** (∼0.70) than **1**–**4** (∼0.51).^61,66^ The stronger *trans* effect of F^–^ than Cl^–^ causes lengthening of the V–N(3) bond (Scheme 7), increasing the tension in the chelate rings. The energy of the compounds **5** and **6**, decreases by adopting trigonal bipyramidal structure in solution. The increase of the trigonality index in 5A and 6A increases the distance between the vanadium atom and N(2), resulting only in five-coordinate species in the solutions of **5** and **6.**(61) The failure to synthesize the V–F compounds with the sterically hindered dbqch and dbqen ligands (the dimethylated molecules) is attributed to the high energy, required for these ligands, to adopt trigonal bipyramidal structure in solution (Scheme 8). The sterically hindered dbqch and dbqen ligands in **2**, and **4**, force N(2) close to the vanadium atom (*vide supra*), taking octahedral or distorted square pyramidal structures only. The low spin - ^19^F superhyperfine coupling constant of **5** and **6** (∼15 × 10^–4^ cm^–1^) than *cis*-[V^IV^(O)(F)(4,4′-dtbipy)_2_]BF_4_ (41 × 10^–4^ cm^–1^),^44^ indicate that the V^IV^–F interactions in **5** and **6** have a much smaller covalent character than the V^IV^–F bond in *cis*-[V^IV^(O)(F)(4,4′-dtbipy)_2_]BF_4_.

**Figure 6:**
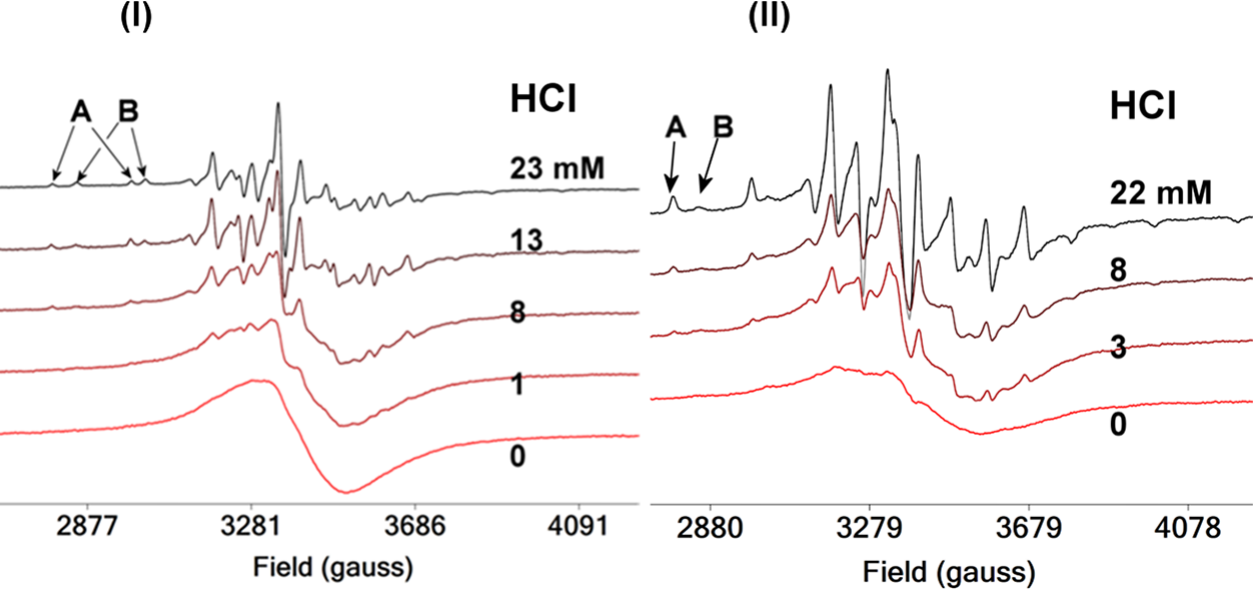
X-band cw EPR spectra of a frozen (120 K) solution (CH_3_CN, 1.25 mM) of the compounds *cis*-[V^IV^(O)(Cl)(dbqen)]Cl·3H_2_O (**A**) (**4·**3H_2_O) and *cis*-[V^IV^(O)(F)(H_2_bqch)]ClO_4_ (B) (**5**) with various quantities of aqueous HCl. The EPR parameters of **4**·3H_2_O and **5** are the same with those of both compounds in frozen DMSO solution.

The X-band cw EPR spectra of the frozen solution of the compounds **1**–**6** in CH_3_CN gave a broad unresolved peak centered at *g* = 1.982 (Figure 6). This spectrum improves with the addition in CH_3_CN of solvents with high dielectric constants such as H_2_O, DMSO etc. In contrast, the X-band EPR spectra of the CH_3_CN solutions at room temperature of **1**–**6** gave well resolved octaplets of both isomers confirming that **A** and **B** are present in CH_3_CN solutions (Figure S9).

Addition of aqueous HCl into the CH_3_CN solution of **4·**3H_2_O (Figure 6I) and **5** (Figure 6II) results in well-resolved spectra that contain both species **A** and **B**. Increasing the quantity of aqueous HCl into the CH_3_CN solution of **1**–**6** the equilibrium is shifted toward **A,** and this is in line with the theoretical calculations (*vide infra*). Extrapolation of the quantities of **A** vs the quantity of aqueous HCl in CH_3_CN shows that both **A** and **B** are present in pure CH_3_CN. The X-band cw EPR spectra of the CH_3_CN solutions of **1**–**6** gave well resolved octuplets of both isomers confirming that **A** and **B** are present in CH_3_CN solutions (Figure S9).

### Speciation of the Catalytic Reaction Mixtures with ^51^V NMR Spectroscopy and 5,5-Dimethylpyrollidone Oxide (DMPO) Trap EPR Experiments

The ^51^V and ^1^H NMR spectra of **3** in solution (CH_3_CN, 5.0 mM) after the addition of H_2_O_2_ (5.0 M, 30%) are shown in Figures S10 and S11 respectively. The ^51^V NMR spectrum of **3** shows the presence of two peaks at −606 ppm and −674 ppm assigned to the monoperoxido and bisperoxido V^V^ species respectively, which are absent from the ^51^V NMR spectrum of an CH_3_CN solution of **3**. Thus, it is obvious that the V^IV^ of compound **3** is oxidized to V^V^ upon addition of H_2_O_2_. Addition of aqueous HCl (50 mM) to the CH_3_CN solution of **3**+H_2_O_2_ results in the appearance of a new peak at −569 ppm (Figure S10) assigned to the dioxido V^V^ and its formation is due to the partial decomposition of peroxido V^V^ complexes. The ^1^H NMR of the ligand at the same conditions and the spike experiments show that the solutions of **3** + H_2_O_2_ and **3** + H_2_O_2_ + aqueous HCl do not contain free ligand (Figure S11). Apparently, the V^V^ peroxido and dioxido species retain the ligands attached to the metal ion.

The cw X-band EPR spectra of **3** in solution (CH_3_CN, 1.0 mM) + H_2_O_2_ (10 mM, 30%) + DMPO (1.0 mM) vs time are shown in Figure S12. After the addition of H_2_O_2_ into the CH_3_CN solution of **3** + DMPO at zero time the EPR spectrum shows a strong peak at *g* = 2.0153 assigned to the radical of DMPO adduct with various radicals that might be formed in solution including superperoxide and hydroxide radicals. This peak after ∼30 min turned to an 9-fold peak at *g* = 2.0044 and *A*_N_ ∼ 7 G and *A*_H_ ∼ 4G identified as 5,5-dimethyl-pyrrolidone-(2)-oxyl- (DMPOX^’^) the oxidation product of DMPO-·OH as assigned previously.^72−74^ In conclusion, the V^IV^ of the catalysts is oxidized to V^V^ upon addition of H_2_O_2_ and the ligands remain bound to the vanadium atom under the conditions of catalysis, whereas, the mechanism of the catalytic reaction is through hydroxyl radicals.

### Mechanistic Details for the Reactivity of the *cis*-[V^IV^(O)(Cl)(N_4_)]^+^ Compounds, with F^–^

**Figure 7:**
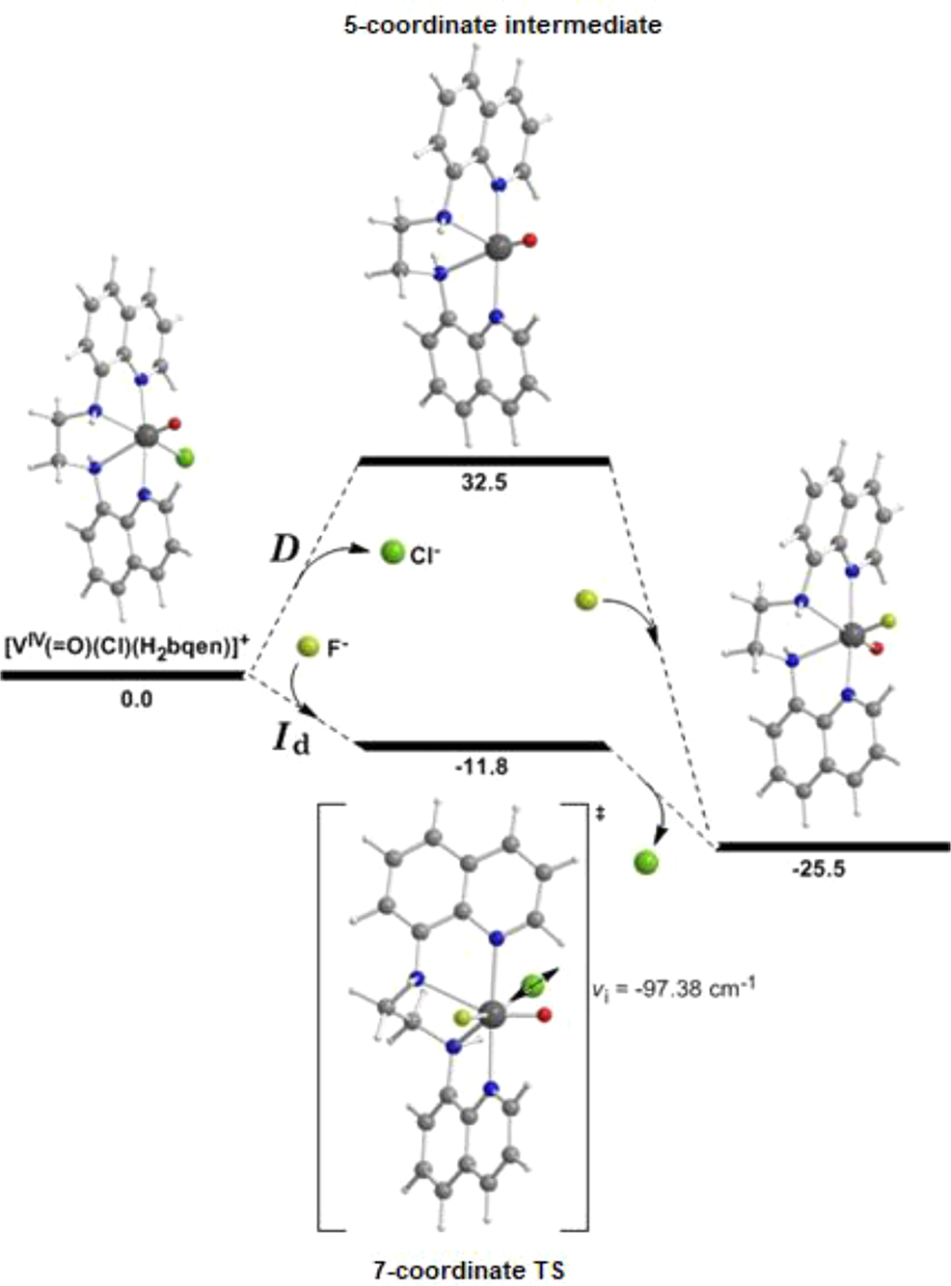
Geometric and energetic profile of the substitution of Cl^–^ by F^–^ in the octahedral *cis*-[V^IV^(O)(Cl)(H_2_bqen)]^+^ complex, following the *D* and *I*_d_ pathways calculated by the PBE0/Def2-TZVP(V)6-31+G(d)(E)/PCM computational protocol in acetonitrile solutions.

The substitution reaction of chloride by fluoride in the *cis*-[V^IV^(O)(Cl)(N_4_)]^+^ (N_4_ = H_2_bqen, H_2_bqch, dbqen, dbqch) compounds was modeled through DFT methods. The dissociative (*D*), associative (*A*) and concerted interchange (both the dissociative *I*_d_ and associative *I*_a_ variations) mechanisms were explored for this reaction. A representative geometric and energetic profile for the ligand substitution reaction of the octahedral *cis*-[V^IV^(O)(Cl)(H_2_bqen)]^+^ is shown in Figure 7. The equilibrium geometries of the *cis*-[V^IV^(O)(Cl)(N_4_)]^+^ (N_4_ = H_2_bqen, H_2_bqch, dbqen, dbqch) compounds between the 5-coordinate [V^IV^(O)(N_4_)]^2+^ and the 7-coordinate [V^IV^(O)(Cl)(F)(N_4_)] transition states in acetonitrile solutions, optimized at the PBE0/Def2-TZVP(V)^υ^6-31+G(d)(E)/PCM level of theory along with selected structural parameters, are given in Figures S13, S14. The optimized structural parameters of the *cis*-[V^IV^(O)(Cl)(N_4_)]^+^ (N_4_ = dbqen, dbqch) and *cis*-[V^IV^(O)(F)(H_2_bqch)]^+^ compounds are in line with those derived from the X-ray structural analysis.

Substitution of Cl^–^ by F^–^ ligand is not reasonable to follow the *I*_a_ mechanism, since all attempts to identify a 7-coordinate transition state or intermediate in these reactions were not successful. The dissociative mechanism (Figure 7) is not favored since the dissociation of Cl^–^ needs relatively high activation energy ∼32.5 kcal/mol for the formation of the 5-coordinate intermediate. It is more likely that the substitution reaction follows the concerted dissociative interchange *I*_d_ pathway. This pathway is “free” of any activation barrier, since the formation of the 7-coordinate transition state releases energy 11.8 kcal/mol with the concerted dissociation of Cl^–^ demanding only 13.7 kcal/mol of energy. The methyl substituents on the amine N atoms of the N_4_ ligand hinder the approach of the incoming F^–^ ligand to attack the vanadium(IV) atom of the compounds to form the 7-coordinate transition state. This fact is in line with the experimental data (vide supra) and explains why our efforts to prepare the *cis*-[V^IV^(O)(F)(N_4,dm_)]^+^ derivatives starting from *cis*-[V^IV^(O)(Cl)(N_4,dm_)]^+^ have failed (N_4,dm_ = the dimethylated derivatives).

### Mechanistic Studies of *cis*-[V^IV^(O)(Cl/F)(N_4_)]^+^ Catalysts through DFT Computations

**Figure 8:**
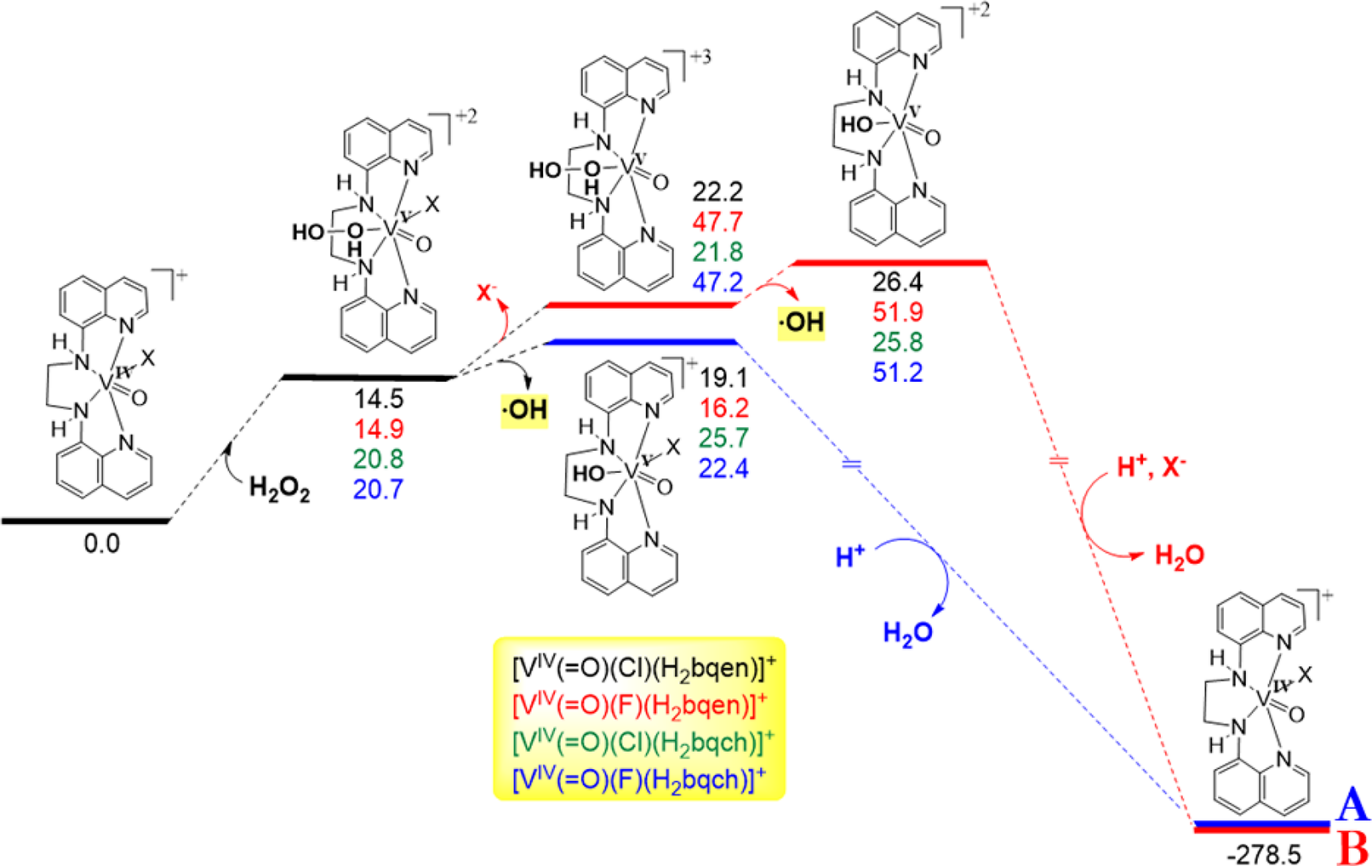
Energetic profiles for the reaction pathways A and B that generate hydroxyl ^•^OH radicals upon homolytic cleavage of the HO–OH bond catalyzed by the *cis*-[V^IV^(O)(X)(N_4_)]^+^ (X = Cl^–^, F^–^; N_4_ = H_2_bqen, H_2_bqch) compounds calculated by the PBE0/Def2-TZVP(V)^υ^6-31+G(d)(E)/PCM computational protocol in acetonitrile solutions.

The oxidation of alkanes catalyzed by vanadium-based catalytic systems proceeds via hydroxyl radicals (^•^OH), generated upon metal catalyzed decomposition of H_2_O_2_, which abstract hydrogen atoms from alkanes (RH) to form alkyl radicals (R^•^).^75^ The energetic profiles for pathways (A and B) that generate ^•^OH radicals upon homolytic cleavage of the HO–OH bond catalyzed by the *cis*-[V^IV^(O)(X)(N_4_)]^+^ (X = Cl^–^, F^–^; N_4_ = H_2_bqen, H_2_bqch) complexes are shown in Figure 8.

The first step in both pathways, A and B, involves the nucleophilic attack on the vanadium metal center by the H_2_O_2_, which is assisted by a hydrogen bond formation O···H–N between the distal O atom of the coordinated H_2_O_2_ and the H atom of the secondary amino moiety. In the methylated catalysts the presence of the methyl groups in the inner coordination sphere of the catalysts hinders the nucleophilic attack on the vanadium atom by the H_2_O_2_, and this is in line with the low yields being 7.7 and 10.2% for **2** and **4** catalysts, respectively. According to NBO analysis, the vanadium central atom acquires positive natural atomic charge ranging from 0.604 up to 0.943 |e|. The natural atomic charge on the vanadium metal center is higher in the fluorido- *cis*-[V^IV^(O)(F)(N_4_)]^+^ than in the chlorido- *cis*-[V^IV^(O)(Cl)(N_4_)]^+^ compounds. Therefore, the *cis*-[V^IV^(O)(F)(N_4_)]^+^ compounds are more susceptible to nucleophilic attack by H_2_O_2_. Interestingly the N donor atoms of the groups -**N**H- and -**N(**CH_3_)- acquire higher negative natural atomic charges (negative natural atomic charges in the range of −0.507 up to −0.682 |e|) than the two quinoline N donor atoms (−0.429 up to −0.457 |e|) of the N_4_ ligand and the X (−0.339 up to −0.541 |e|) and O (−0.417 up to −0.501 |e|) donor atoms of the catalysts.

In the reaction pathway A, the second step involves the homolytic cleavage of the O–O bond in the [V^V^(O)(H_2_O_2_)(X)(N_4_)]^2+^ (X = F^–^, Cl^–^) species generating directly ^•^OH radicals and the (oxido)(hydroxido) [V^V^(O)(OH)(X)(N_4_)]^+^ species. The estimated energy barriers for the generation of the ^•^OH radicals are 19.1, 16.2, 25.7, and 22.4 kcal/mol for the [V^V^(O)(H_2_O_2_)(Cl)(H_2_bqen)]^2+^, [V^V^(O)(H_2_O_2_)(F)(H_2_bqen)]^2+^, [V^V^(O)(H_2_O_2_)(Cl)(H_2_bqch)]^2+^, and [V^V^(O)(H_2_O_2_)(F)(H_2_bqch)]^2+^ species, respectively. According to the estimated energy barriers for the [V^V^(O)(H_2_O_2_)(X)(H_2_bqen)]^2+^ and [V^V^(O)(H_2_O_2_)(X)(H_2_bqch)]^2+^ species, the catalytic efficacy of the former should be higher than the latter. This is in line with the experimental catalytic activity of the fluorine vanadium compounds, **5** and **6**. The chloride (X = Cl^–^) exhibits the same catalytic activity, and this can be interpreted if we assume that Cl^–^ compounds follow an alternative mechanism, pathway B (*vide infra*). The resulting from the homolytic cleavage of the O–O bond [V^V^(O)(OH)(X)(N_4_)]^+^ species reacts with protons and yields the (oxido)(aquo) [V^iV^(O)(OH_2_)(X)(N_4_)]^+^ species (Figure 8) which releases directly the aquo ligand to regenerate the *cis*-[V^IV^(O)(X)(N_4_)]^+^ catalysts.

In the reaction pathway B (Figure 8), the second step involves coordination of H_2_O_2_ nucleophile to the vanadium atom promoting the dissociation of the leaving ligand X (X = F^–^, Cl^–^) yielding the transient *cis*-[V^V^(O)(O_2_H_2_)(H_2_bqen)]^3+^ and *cis*-[V^V^(O)(O_2_H_2_)(H_2_bqch)]^3+^ species. The third step along the reaction pathway B involves the homolytic cleavage of the O–O bond in the *cis*-[V^V^(O)(O_2_H_2_)(N_4_)]^3+^ (N_4_ = H_2_bqen, H_2_bqch) species yielding the *cis*-[V^V^(O)(OH)(H_2_bqen)]^2+^ cation. The homolytic cleavage of the HO–OH bond, of the vanadium coordinated H_2_O_2_, demands very low energy (around 4.5 kcal/mol), while the energy of the homolytic cleavage of the “free” H_2_O_2_ is 44.1 kcal/mol at the PBE0/6-31+G(d)(E)/PCM level of theory. Next, the *cis*-[V^IV^(O)(OH)(N_4_)]^+^ species reacts with protons and Cl^–^ with concomitant release of the aquo ligand to regenerate the [V^IV^(O)(X)(N_4_)]^+^ catalysts.

The breaking of the V–F and V–Cl bonds demands an energy barrier 48 and 22 kcal/mol, respectively. The high energy barriers for breaking the V–F bonds are not in favor of the reaction pathway B for the fluorine V^IV^ species. Pathway A predicts the catalytic activity of flourido- vanadium complexes. Unlikely, pathway B predicts chloride- complexes to have higher activity than the flourido- complexes and **5** and **6** to exhibit the same activity. On the other hand, the chloride complexes **1** and **3** follow pathway B, predicting both compounds to exhibit similar catalytic activity in line with the experiment. The energy barriers for the [V^V^(O)(H_2_O_2_)(H_2_bqen)]^3+^ and [V^V^(O)(H_2_O_2_)(H_2_bqch)]^3+^ species are almost the same.

Apparently, the oxidation of alkanes catalyzed by vanadium-based catalytic systems proceeds through pathway A for the fluoride/vanadium and through pathway B for the chloride/vanadium compounds. The reaction pathway is controlled by the strength of the V–X (X = F^–^, Cl^–^) bond. The lower activity of the N–CH_3_ than N–H vanadium compounds is attributed to the steric hindrance caused by the methyl groups, hindering H_2_O_2_ approach to vanadium at the first step of the reaction.

### Mechanistic Details for the Catalytic Oxidation Reactions with Addition of HCl

The catalytic activity of the oxidovanadium(IV) compounds toward cyclohexane oxidation in the presence of HCl was also investigated by DFT calculations on the protonated at the -**N(**CH_3_)- moieties of the [V^IV^(O)(Cl)(N_4_H^+^)]^2+^ (N_4_H^+^ = dbqenH^+^, dbqchH^+^) compounds. The equilibrium geometries of all species and products involved in the reaction pathways that yield the hydroxyl ^•^OH catalyzed by the protonated methylated [V^IV^(O)(X)(N_4_H)]^2+^ (X = Cl^–^, F^–^; N_4_H^+^ = dbqenH^+^, dbqchH^+^) catalysts optimized at the PBE0/Def2-TZVP(V)^υ^6-31+G(d)(E)/PCM level of theory in acetonitrile solutions, along with selected structural parameters are given in Figure S15.

**Figure 9:**
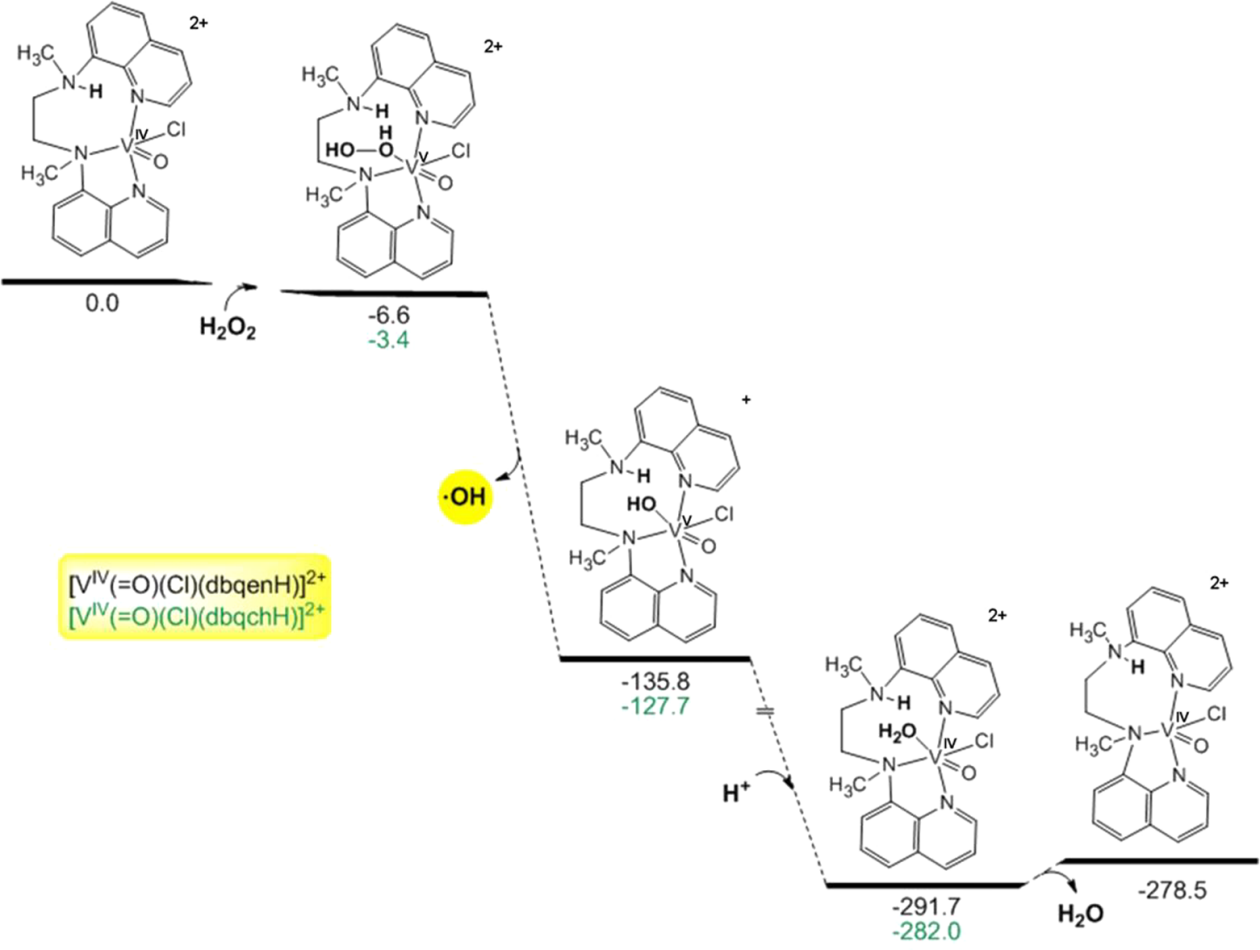
Energetic profile for the reaction pathway that generates hydroxyl ^•^OH radicals catalyzed by the [V^IV^(O)(Cl)(N_4_H)]^2+^ (N_4_H^+^ = dbqenH^+^, dbqchH^+^) compounds calculated by the PBE0/Def2-TZVP(V)^υ^6-31+G(d)(E)/PCM computational protocol in acetonitrile solutions.

The protonation of -**N(**CH_3_)- induces remarkable changes on the inner coordination sphere of the vanadium (Figure 9), more specifically: (i) its coordination number changes from six to five, in line with the EPR experiment, and thus, leaving an open site for the coordination of H_2_O_2_ to vanadium atom. ιi) the amine hydrogen atom of [-N**H**(CH_3_)-]^+^ assists the H_2_O_2_ attack to the vanadium atom by the formation of a hydrogen bond between the distal O atom of the coordinated O_2_H_2_ and the amine hydrogen atom of the [N**H**(CH_3_)]^+^ moiety.

The formation of the [V^V^(O)(H_2_O_2_)(Cl)(dbqenH^+^)]^3+^ species is slightly exothermic (Δ*H* = −4.3 kcal/mol), while the formation of the [V^V^(O)(H_2_O_2_)(Cl)(dbqchH^+^)]^3+^ species is slightly endothermic (Δ*H* = 3.4 kcal/mol).

The second step involves the homolytic cleavage of the O–O bond in the [V^V^(O)(H_2_O_2_)(Cl)(N_4_H^+^)]^3+^ (N_4_H^+^ = dbqenH^+^, dbqchH^+^) species generating directly ^•^OH radicals and [V^V^(O)(OH)(Cl)(N_4_H^+^)]^2+^ species, through a strongly exothermic dissociation process (Δ*H* ∼ −128 up to −131 kcal/mol). Next, the [V^V^(O)(OH)(Cl)(N_4_H^+^)]^2+^ cation reacts with protons (HCl) and yields the (oxido)(aquo) [V^IV^(O)(OH_2_)(Cl)(N_4_H^+^)]^2+^ species (Figure 9) which releases the aquo ligand and the catalysts [V^IV^(O)(Cl)(N_4_H^+^)]^2+^. The transformation of [V^V^(O)(OH)(Cl)(N_4_H^+^)]^2+^ cation to [V^IV^(O)(Cl)(N_4_H^+^)]^2+^ species in the acidic media is strongly exothermic (Δ*H* ∼ −143 up to −156 kcal/mol).

The pathway in Figure 9 agrees with the experimental efficiency toward the catalytic oxidation of alkanes observed for all the complexes **1**–**6** in the presence of HCl.

## Conclusions

In summary, a series of four oxidovanadium(IV) compounds of the general formula *cis*-[V^IV^(O)(Cl)(N_4_)]Cl was prepared by reacting V^IV^OCl_2_ with the nonplanar tetradentate N_4_ bis-quinoline ligands. Sequential treatment of the two nonmethylated N_4_ oxidovanadium(IV) compounds with KF and NaClO_4_ resulted in the isolation of the species with the general formula *cis*-[V^IV^(O)(F)(N_4_)]ClO_4_. The oxidovanadium(IV) compounds were physicochemically and structurally characterized.

Their catalytic oxidation reactions of the highly distorted octahedral V^IV^O^2+^ compounds, mimicking the irregular geometries of the coordination environment of the metal ions in proteins, with the nonplanar N_4_ tetradentate amine ligands were examined. The distortion of the coordination sphere of the V^IV^O^2+^ cation induced by the N_4_ ligands was further enforced by partially replacing ligand’s H- with bulky cyclohexyl- and/or methyl- groups and by introducing F- or Cl- coligands in the V^IV^O^2+^ coordination sphere.

The experimental EPR parameters of these distorted V^IV^O^2+^ compounds deviate from those calculated from the empirical additivity relationship. The deviation has been assigned either to the coordination of the axial nitrogen donor atom or the trigonal distortion of the V^IV^ coordination environment. cw X-band EPR speciation studies in frozen polar solvents reveal that the introduction of the hindered cyclohexyl- and methyl- groups causes retention in solution of the octahedral solid-state crystal structure, whereas, ligands without steric hindrance allow dissociation of one of the ligand’s amine donor atom from the six-coordinate sphere of V^IV^ ion in solution, resulting in five-coordinate structures. Based on the equilibrium between six- and five-coordinate species, we concluded that the steric hindrance in the V^IV^O^2+^ compounds is increasing according to the following series, -HNCH_2_CH_2_NH- > -HNC_6_H_10_NH- > -(CH_3_)NCH_2_CH_2_N(CH_3_)- > -(CH_3_)NC_6_H_10_N(CH_3_)-. cw X-band EPR spectra of the V^IV^O^2+^ compounds in frozen CH_3_CN show that **1**–**6** five- or both five and six-coordinate structures, however addition of aqueous HCl into their CH_3_CN solution results in the full dissociation of the equatorial amine group and the formation of only five-coordinate species. The sterically hindered compounds **2** and **4**, containing the dimethylated ligands, inhibit the approach of the nucleophiles (F^–^, H_2_O_2_) to the vanadium nucleus, resulting in unsuccessful replacement of Cl^–^ ligand by the F^–^ and lower oxidative catalytic activity compared with the less sterically hindered **1** and **3**, which contain the nonmethylated ligands.

The variation of the oxidative catalytic activities between the chloride and fluoride V^IV^ compounds is attributed to two different mechanisms of catalytic action controlled by the V-X (X = F^–^, Cl^–^) bond strengths (V–F is stronger than V–Cl). The generation of ^•^OH radical for the *cis*-[V^IV^(O)(**Cl**)(N_4_)]^+^ species takes place via the dissociation of Cl^–^, while for the *cis*-[V^IV^(O)(**F**)(N_4_)]^+^ species via the formation of seven-coordinate [V^IV^(O)(**F**)(H_2_O_2_)(N_4_)]^+^ cation. The distortion of the coordination environment of the V^IV^ ion, mimicking the active site of metal-proteins, can be used as a highly desirable methodology allowing for the modification of the functionality of the metal compounds such as in the case of oxidative catalysis.

The vanadium(IV) of the compounds **1**–**6** is oxidized to vanadium(V) upon addition of H_2_O_2_ and the ligands remain bound to the vanadium atom under the conditions of catalysis, as it was evidenced with ^51^V and ^1^H NMR spectroscopies. cw X-band EPR trap studies proved that the mechanism of the catalytic reaction is through hydroxyl radicals.

Suitable ligands that introduce the desirable amount of distortion on the metal ion’s coordination environment can result in a fruitful design approach for the development of effective catalysts tailored for specific applications.

## Supporting Information Available

UV–vis-NIR, FT-IR, NMR spectra, and crystallographic data (PDF)
