# Supplementary material for: A Combined Experimental and Theoretical Investigation of Oxidation Catalysis by cis-[VIV(O)(Cl/F)(N4)]+ Species Mimicking the Active Center of Metal-Enzymes
Source: Inorg Chem. 2022 Nov 10;61(46):18434–49. doi: 10.1021/acs.inorgchem.2c02526 (PMC9682486; doi:10.1021/acs.inorgchem.2c02526)
Supplement: Supplementary file 1 — ic2c02526_si_001.pdf [file ic2c02526_si_001.pdf]

Supporting Information

A Combined Experimental and Theoretical Investigation of Oxidation Catalysis by *cis*-$[V^{IV}(O)(Cl/F)(N_4)]^+$ Species Mimicking the Active Center of Metal-Enzymes

Michael G. Papanikolaou,[†,‖] Anastasia V. Simaioforidou,[†] Chryssoula Drouza,[§] Athanassios C. Tsipis,[*,†] Haralampos N. Miras,[*,⊥] Anastasios D. Keramidas,[*,‖] Maria Louloudi,[*,†] and Themistoklis A. Kabanos[*,†]

[†]Section of Inorganic and Analytical Chemistry, Department of Chemistry, University of Ioannina, Ioannina 45110, Greece

[§]Department of Agricultural Production, Biotechnology and Food Science, Cyprus University of Technology, 3036 Limasol, Cyprus

[⊥]West CHEM, School of Chemistry, University of Glasgow, Glasgow G12 8QQ, U.K.

[‖]Department of Chemistry, University of Cyprus, Nicosia 1678, Cyprus

Contents

Pages

Experimental 4

Figure S1. IR spectrum of compound **1**. 7

Figure S2. IR spectrum of compound **2**. 8

Figure S3. IR spectrum of compound **3**. 9

Figure S4. IR spectrum of compound **4**. 10

Figure S5. IR spectrum of compound **5**. 11

Figure S6. IR spectrum of compound **6**. 12

Figure S7. X-band cw EPR spectrum of a frozen solution of the compound *cis*-$[V^{IV}(O)(F)(H_2bqch)]ClO_4$ **(5)** in DMSO (1.00 mM) at 120 K and its simulated spectrum.13

Figure S8. X-band cw EPR spectrum of a frozen solution of the compounds **1-6** in DMSO (1.00 mM) at 120 K. 14

Figure S9. X-band cw EPR spectrum of DMSO and CH_3CN solutions of **2** at RT and at 120 K. 15

Figure S10. The ^{51}V spectra of an CH_3CN solution of **3** (5.0 mM) a) after the addition of H_2O_2 (5.0 M, 30 %) (black line) and b) after the addition of H_2O_2 (5.0 M, 30 %) and 50mM HCl. 16

Figure S11. The ^{1}H spectra of an CH_3CN solution of a) **3** (2.0 mM) after the addition of H_2O_2 (2.0 M, 30 %) (black line) and b) H_2bqen (2.0 mM) after the addition of H_2O_2 (2.0 M, 30%). 17

Figure S12. cw X-band EPR spectra of CH_3CN solution of **3** (1.0 mM) + H_2O_2 (10 mM, 30%) + DMPO (1.0 mM) *vs* time in min. 18

Figure S13. Equilibrium geometries of the *cis*-$[V(=O)(Cl)(N_4)]^+$ (N_4 = H_2bqen, H_2bqch, dbqen, dbqch) compounds in acetonitrile solutions optimized at the PBE0/Def2-TZVP(V)□6-31+G(d)(E)/PCM level of theory. 19

19

Figure S14. Equilibrium geometries of the 5-coordinate $[V(=O)(N_4)]^{2+}$ species and the 7-coordinate $[V(=O)(Cl)(F)(N_4)]$ transition states in acetonitrile solutions optimized at the PBE0/Def2- TZVP(V)υ6-31+G(d)(E)/PCM level of theory. 20

Figure S15. Equilibrium geometries of the $[V(=O)(Cl)(N_4H^+)]^{2+}$, $[V(=O)(Cl)(H_2O_2)(N_4H^+)]^{2+}$ and $[V(=O)(Cl)(OH)(N_4H^+)]^{2+}$ (N_4 = dbqenH$^+$, dbqchH$^+$) species in acetonitrile solution optimized at the PBE0/Def2-TZVP(V)υ6-31+G(d)(E)/PCM level of theory. 21

Table S1. Crystal data and details of the structure determination and refinement for compound **2’**. 22

Table S2. Interatomic Distances (Å) and Angles (deg) Relevant to the Vanadium Coordination Sphere for compound **2’**. 23

Experimental

Materials, Syntheses, and Physical Measurements. All chemicals were bought from Sigma-Aldrich, were the best available purity and used without further purification unless otherwise stated. C, H, and N analyses were conducted by the microanalytical service of the School of Chemistry, the University of Glasgow; vanadium was determined by atomic absorption. Compounds **5** and **6** were analyzed gravimetrically (as $Pb^{II}ClF$)[1] for fluoride. Chloride was determined gravimetrically as AgCl. Tetrahydrofuran was dried by prolonged reflux under an argon atmosphere over sodium wire with a benzophenone ketyl indicator and it was distilled just prior to use. The dimethylated ligands dbqch and dbqen and the oxidovanadium(IV) compounds were prepared under an argon atmosphere using standard Schlenk techniques. Solid state magnetic susceptibilities were measured at room temperature using a magnetic balance based on the Gouy method. Data were corrected for the intrinsic underlying diamagnetism of the sample using Pascal's constants.[2] Merck silica gel 60 F_{254} TLC plates were used for thin layer chromatography.

Perchlorates are powerful oxidizers, they are potentially hazardous, especially in contact with reducing material and they may explode when exposed to shock or heat.[3]

EPR Spectroscopy. The cw X-band EPR spectra of DMSO solutions of the complexes at 120 K were measured on an ELEXSYS E500 Bruker spectrometer at resonance frequency ~9.8 GHz and modulation frequency 100 MHz. The resonance frequency was accurately measured with solid DPPH (g=2.0036). Simulations of EPR spectra were performed using Easyspin.[4] MultiSpecEPR was used for the graphics. (https://sourceforge.net/projects/multispecepr/).

NMR Spectroscopy. NMR spectra were recorded on a Bruker Avance 500 spectrometer at 500 MHz for ^{1}H and 131.75 MHz for ^{51}V. A 30°-pulse width, 3000 Hz spectral window, 1 s

relaxation delay and a 90° -pulse width, 25000 Hz spectral window, 0.1 s relaxation delay was applied for ^{1}H and ^{51}V NMR respectively. The samples were prepared from purified molecules in CD_3CN at room temperature just prior to NMR spectrometric measurements. Data acquisition and processing were accomplished using TopSpin 4.0.6 and MultiSpecNMR 4.0.0 (https://sourceforge.net/projects/multispecnmr/). Standard pulse programs as implemented in TopSpin were used for data acquisition.

Conductivity. The conductivity was measured in 1mM CH_3CN and DMSO solutions of the complexes at 23°C using a pH/mV/Cond./TDS/Temp. Meter 86505 (MRC Scientifc Instruments). A 0.02M KCl solution was used as a standard solution (2.659 mS at 23°C).

X-ray Crystallography. X-ray diffraction data of single crystals of the **2'**, **2''**, **4'**, **5'** and **6'** compounds were collected by means of a Xcalibur Oxford diffractometer equipped with a Sapphire 3 CCD detector and a 4-cycle Kappa geometry goniometer, using enhanced Mo $K\alpha$ (λ = 0.71073 Å) X-ray source and graphite radiation monochromator. The structure of the compounds was solved by direct methods and refined by full-matrix least-squares techniques on F^2 by using SHELXS-97[5-6]. Special computing molecular graphics incorporated in the WinGX 3.2 interface were used[7]. All the non-H atoms were anisotropically refined. The positions of hydrogen atoms in all structures were calculated from stereochemical considerations and kept fixed isotropic during refinement or found in DF map and refined with isotropic thermal parameters.

Catalysis. All substrates were purchased from Aldrich, in their highest commercial purity, stored at 5 °C and passed through a column containing active alumina to remove peroxide impurities. A 30% aqueous solution of hydrogen peroxide was used as primary oxidant. GC analysis was performed using a Shimadzu GC-17A gas chromatograph coupled with a GCMS-QP5000 mass spectrometer using a SPB-5 column (30 m, 0.25 mm i.d, 0.25 μm).

Chromatography grade helium was used as the carrier gas. The temperature program for cyclohexane was: 50 °C for the first 5 min, followed by a 10 °C min^{-1} temperature gradient to 200 °C for 5 min. Chromatography grade helium was used as the carrier gas, linear velocity= 36.8 cm s^{-1} at column pressure 64.5 kPa.

The catalytic tests were carried out at room temperature (25 ±0.5 °C) by using the following molar ratio [catalyst : H_2O_2 : cyclohexane] = [1:1000:2500 μmoles] with or without 100 μmoles of HCl. 1 μmol of oxidovanadium(IV) complex was diluted in 1ml of CH_3CN; then 100 μmoles of HCl (optional), 1 mmol of cyclohexane, and 2.5 mmol of H_2O_2 were added. The reactions were completed within 6 h. Since the formation of alkyl hydroperoxides was expected, the sample was treated with PPh_3 according to Shul'pins' procedure[8] followed by the addition of 1mmol of bromobenzene as internal standard. Quantitative GC-analysis provided the substrate conversion and product yield by comparing the integrals of their GC peaks *vs.* the internal standard integral. To ensure the identity of oxidation products, their retention time and mass spectral data were compared to those of commercially available products. Blank experiments showed that without oxidovanadium(IV) complex oxidation reactions do not occur.

Computational details. All density functional theory (DFT) calculations were performed using the Gaussian09, D.01 program suite.[9] The geometries of the reactants, intermediates, transition states, catalytic species and products were fully optimized, employing the Perdew, Burke and Ernzerhof,[10-16] PBE0 (also called PBE1PBE) hybrid density functional. For vanadium and main group elements of the complexes under consideration the Def2-TZVP[17-18] and 6-31+G(d) basis sets were used respectively. Hereafter the DFT computational protocol used is abbreviated as PBE0/Def2-TZVP(V)∪6-31+G(d)(E) (E=main group element). The stationary points were identified as local minima by the absence of imaginary

frequencies (N_{Imag} = 0). Solvent effects were taken into account employing the polarizable continuum model (PCM) using the integral equation formalism variant (IEFPCM) being the default self-consistent reaction field (SCRF) method.[19] The natural bond orbital (NBO) population analysis was performed using Weinhold's methodology as implemented in the NBO 6.0 software.[20-22]

IR Spectroscopy

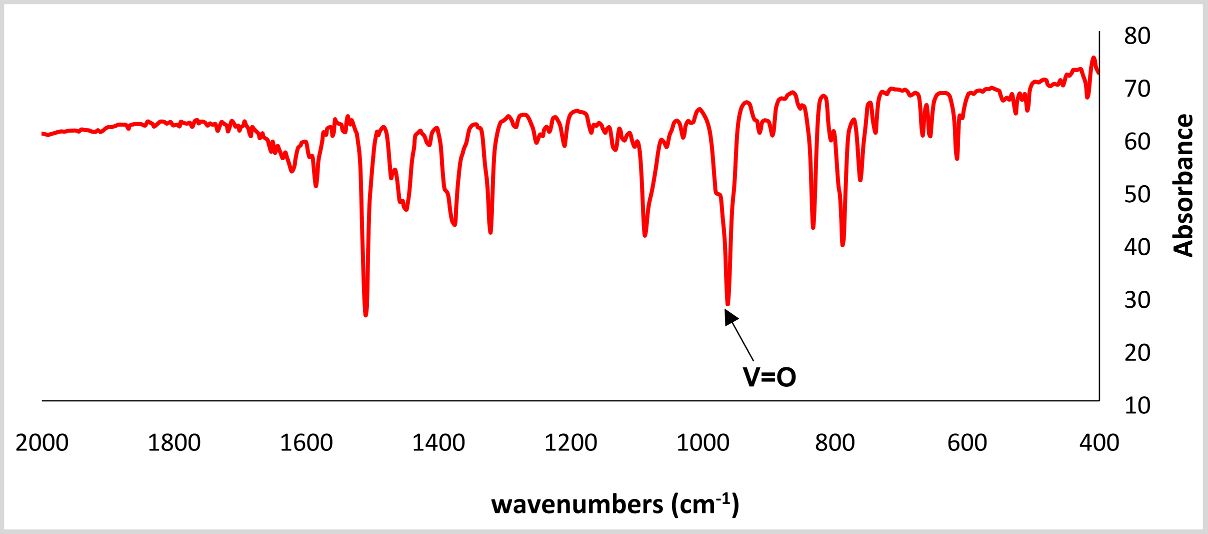

Figure S1. IR spectrum of compound **1**.

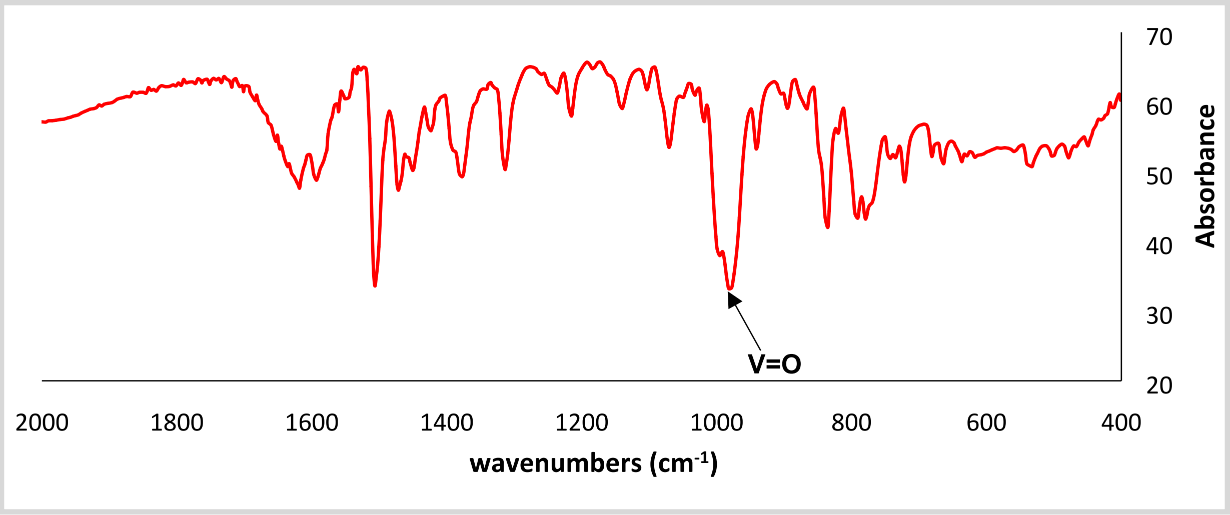

Figure S2. IR spectrum of compound **2**.

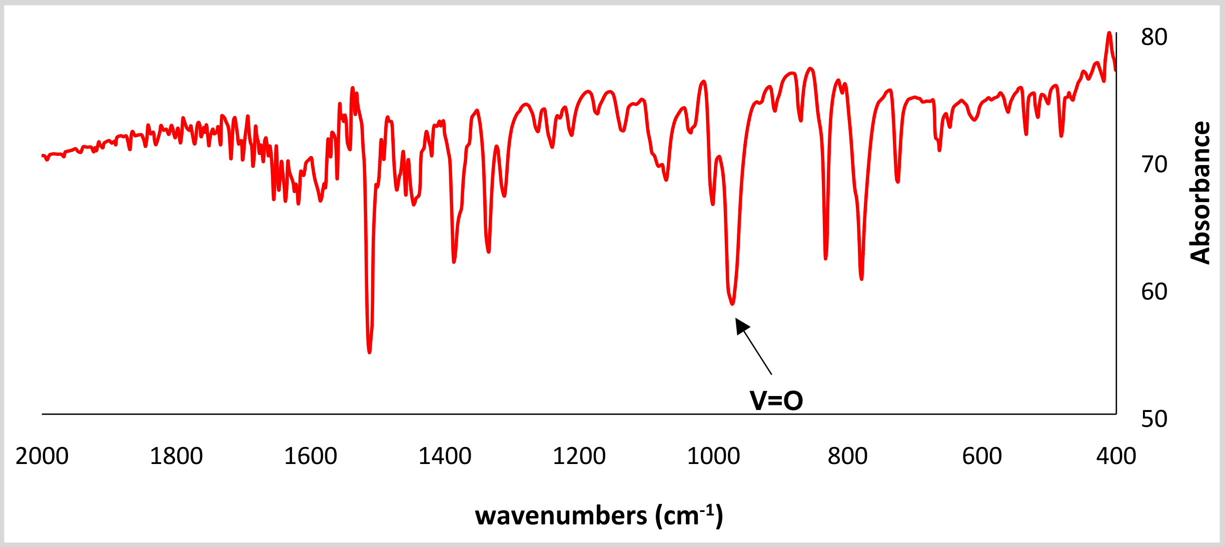

Figure S3. IR spectrum of compound **3**.

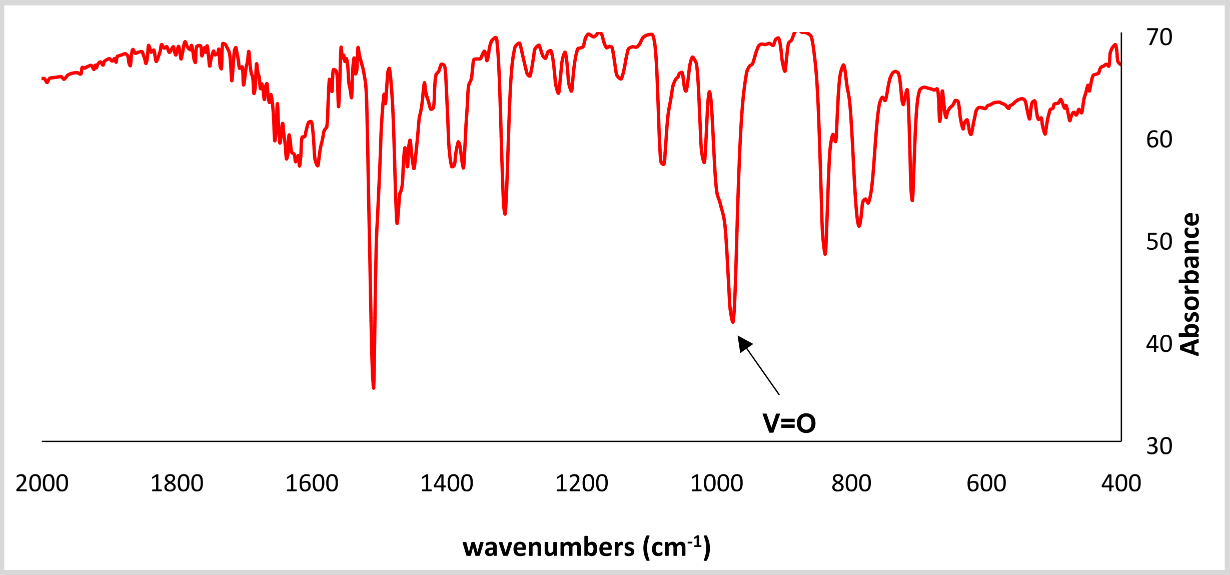

Figure S4. IR spectrum of compound **4**.

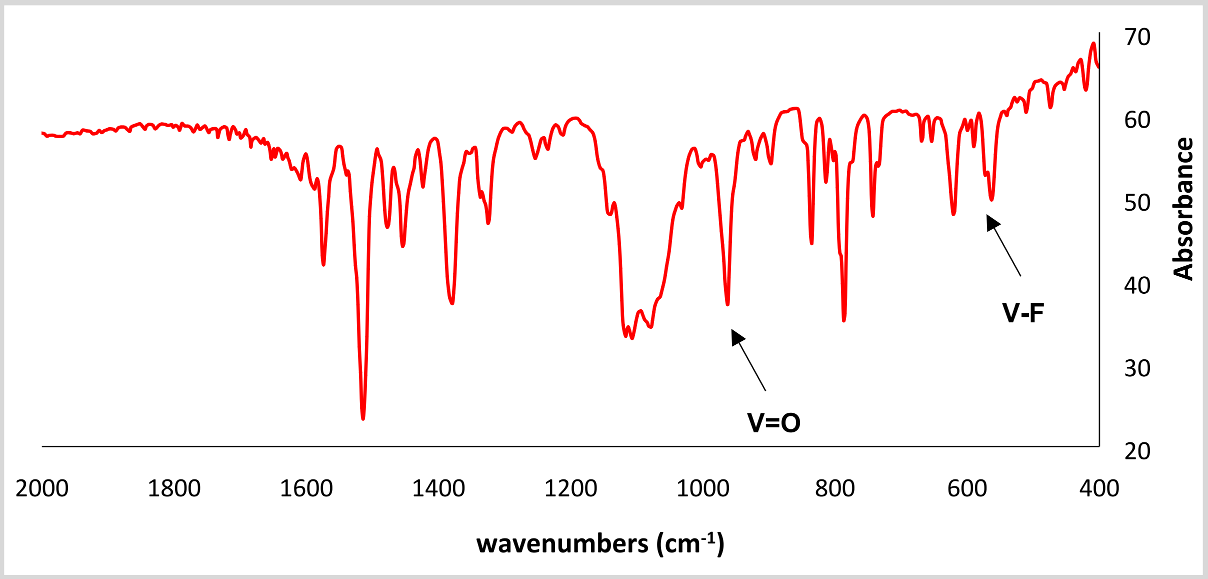

Figure S5. IR spectrum of compound **5**.

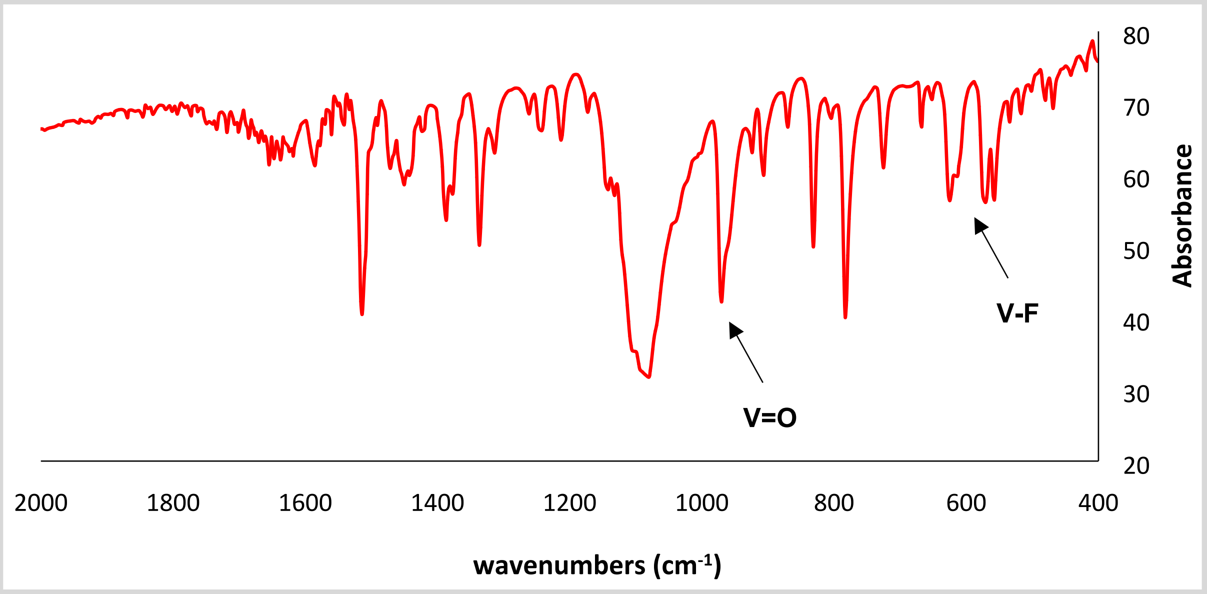

Figure S6. IR spectrum of compound **6**.

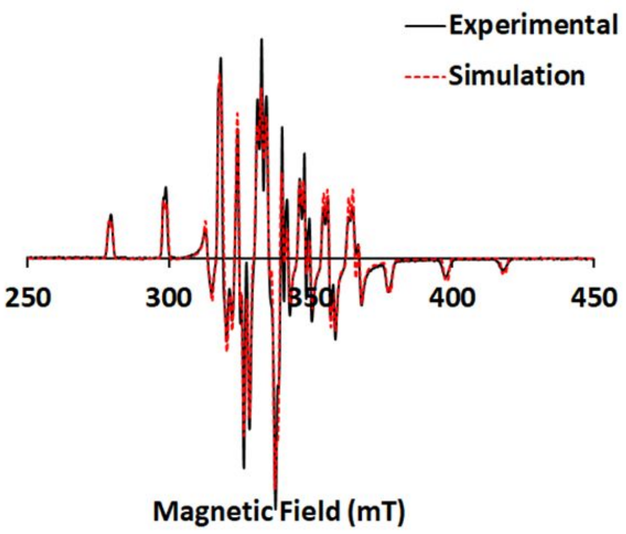

Figure S7. X-band cw EPR spectrum of a frozen solution of the compound *cis*-[V^{IV}(O)(F)(H_2bqch)]ClO_4 **(5)** in DMSO (1.00 mM) at 120 K and its simulated spectrum.

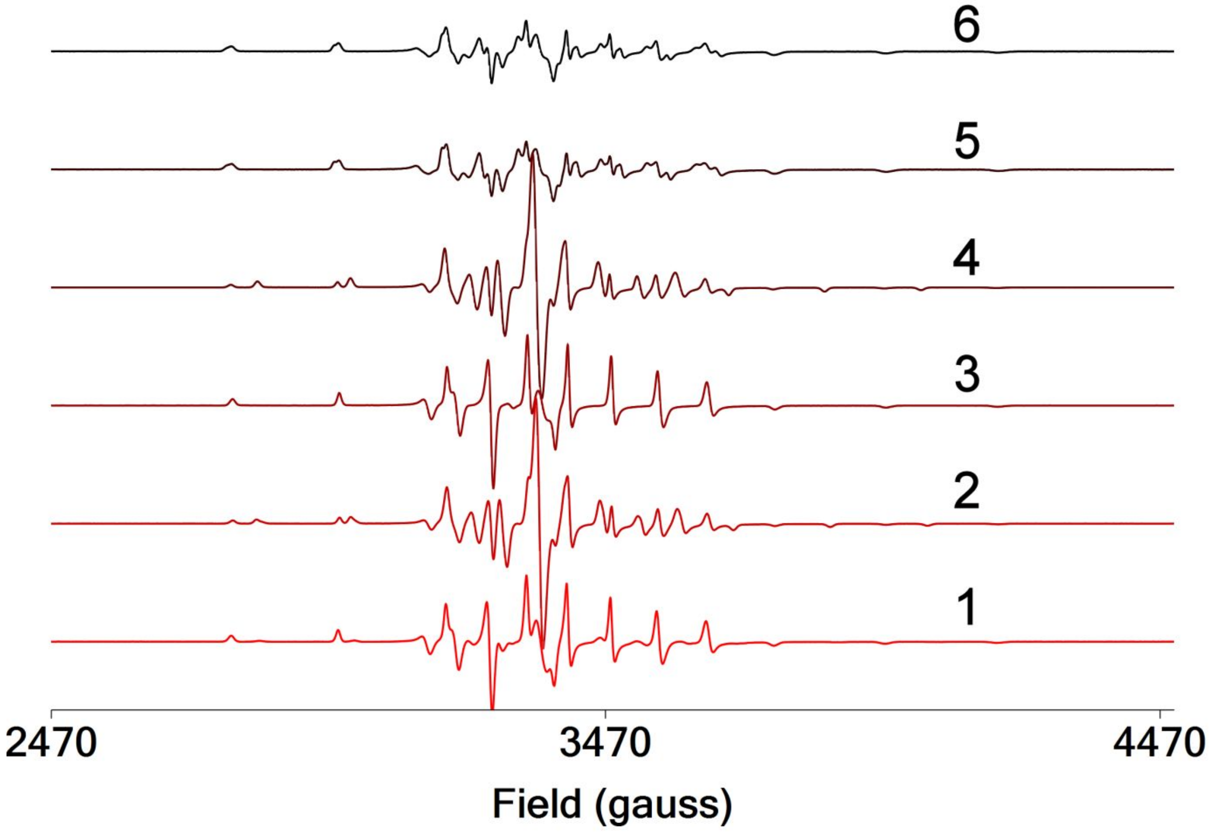

Figure S8. X-band cw EPR spectrum of a frozen solution of the compounds **1-6** in DMSO (1.00 mM) at 120 K.

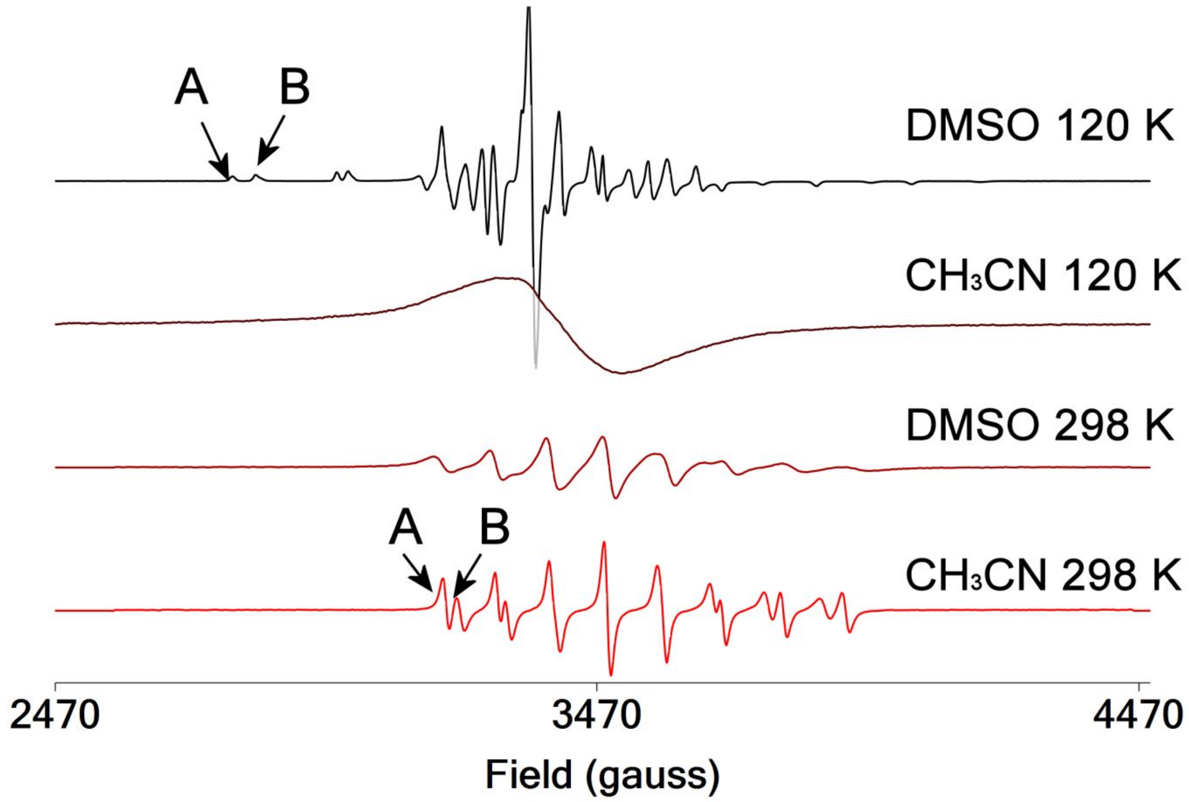

Figure S9. X-band cw EPR spectra of DMSO and CH_3CN solutions of **2** at RT and at 120 K.

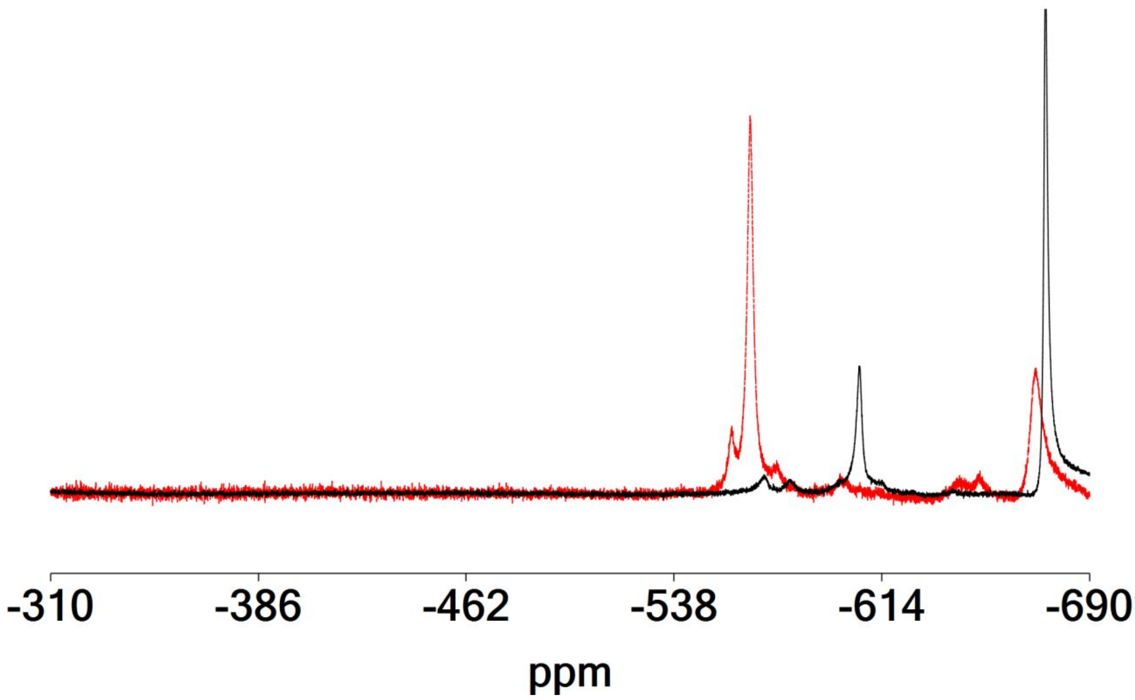

Figure S10. The ^{51}V spectra of an CH_3CN solution of **3** (5.0 mM) a) after the addition of H_2O_2 (5.0 M, 30 %) (black line) and b) after the addition of H_2O_2 (5.0 M, 30 %) and 50mM HCl.

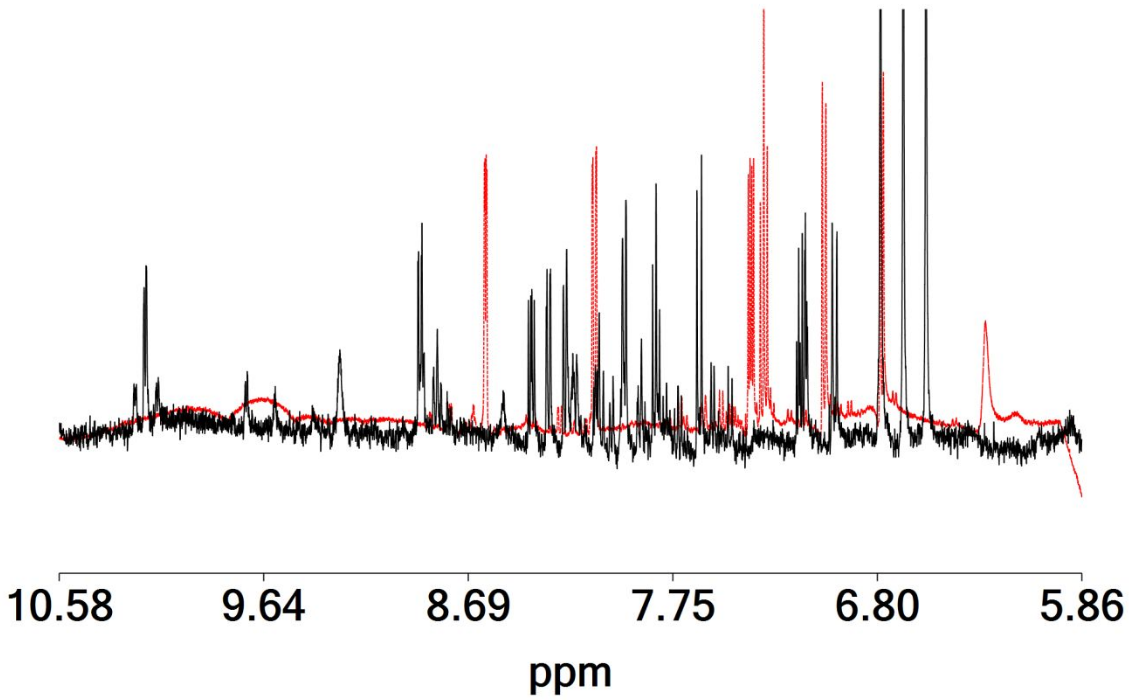

Figure S11. The ^{1}H spectra of an CH_3CN solution of a) **3** (2.0 mM) after the addition of H_2O_2 (2.0 M, 30 %) (black line) and b) H_2bqen (2.0 mM) after the addition of H_2O_2 (2.0 M, 30%).

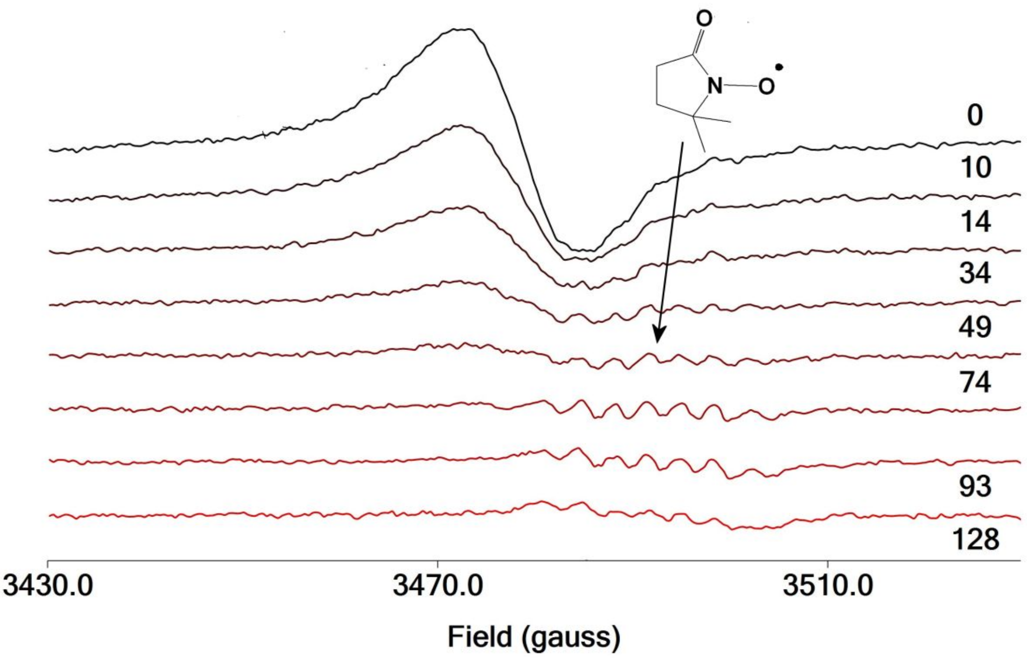

Figure S12. cw X-band EPR spectra of CH_3CN solution of **3** (1.0 mM) + H_2O_2 (10 mM, 30%) + DMPO (1.0 mM) *vs* time in min.

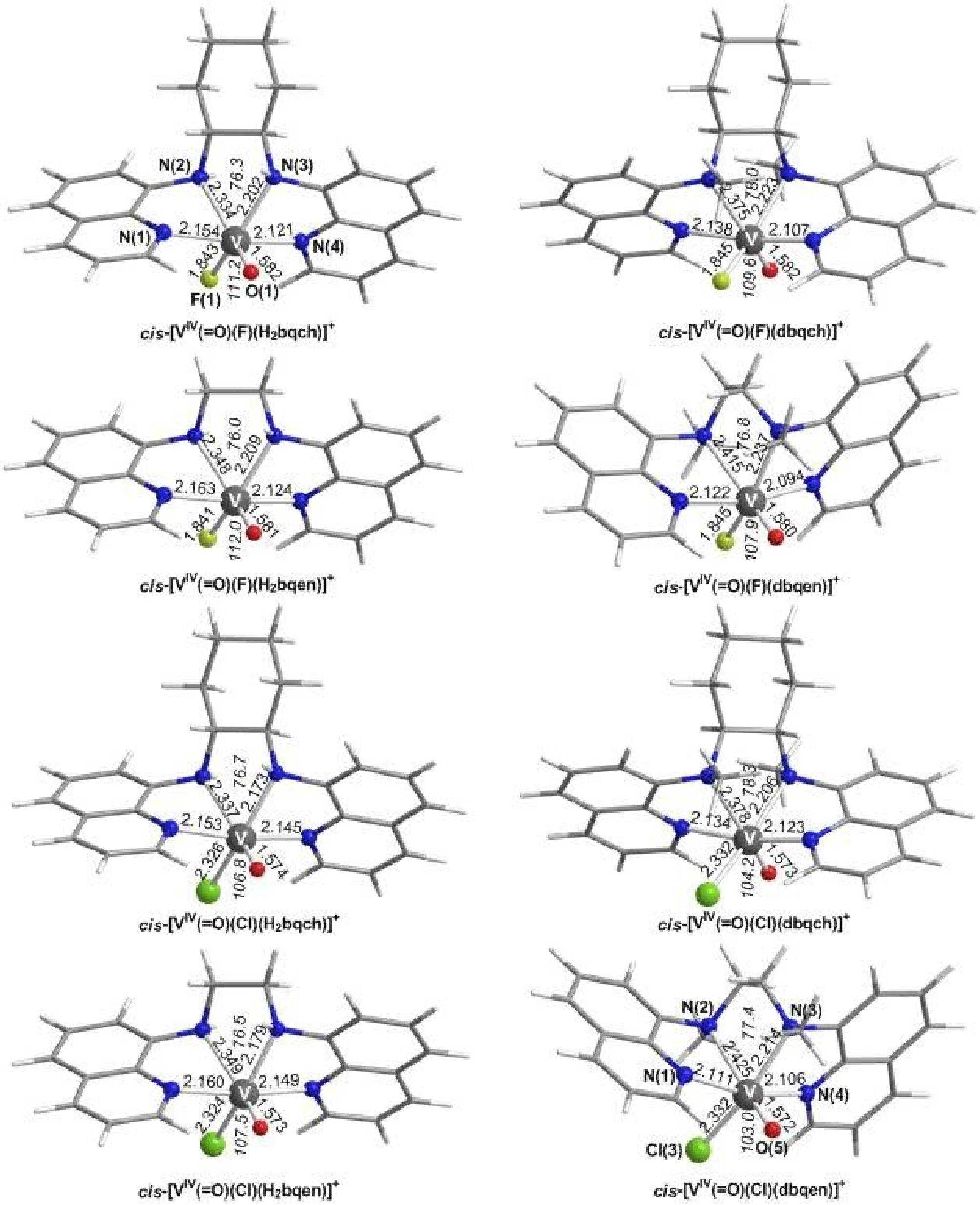

Figure S13. Equilibrium geometries of the *cis*-$[V(=O)(Cl)(N_4)]^+$ (N_4 = H_2bqen, H_2bqch, dbqen, dbqch) complexes in acetonitrile solutions optimized at the PBE0/Def2-TZVP(V)∪6-31+G(d)(E)/PCM level of theory.

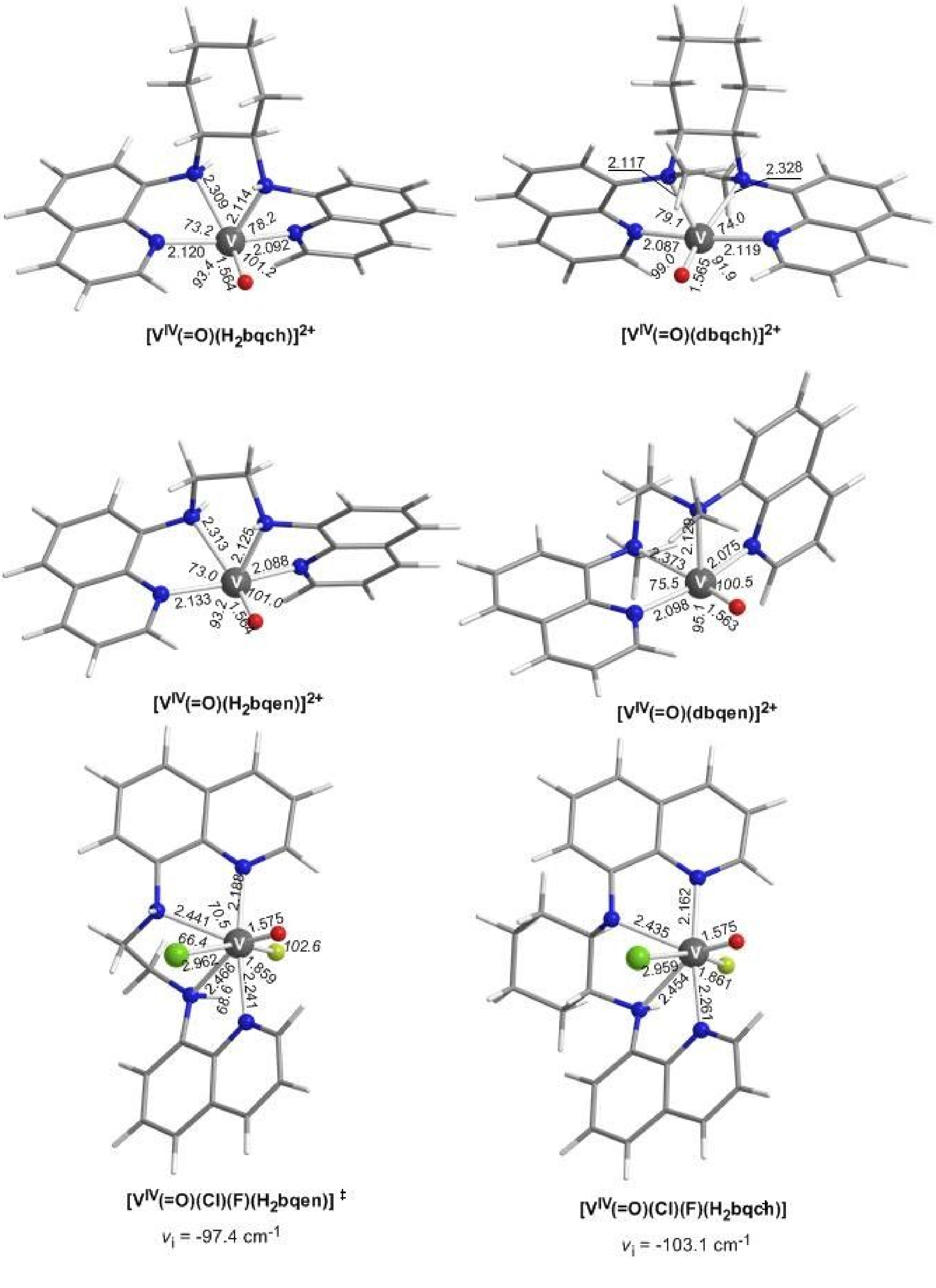

Figure S14. Equilibrium geometries of the 5-coordinate $[V(=O)(N_4)]^{2+}$ species and the 7-coordinate $[V(=O)(Cl)(F)(N_4)]$ transition states in acetonitrile solutions optimized at the PBE0/Def2- TZVP(V)∪6-31+G(d)(E)/PCM level of theory.

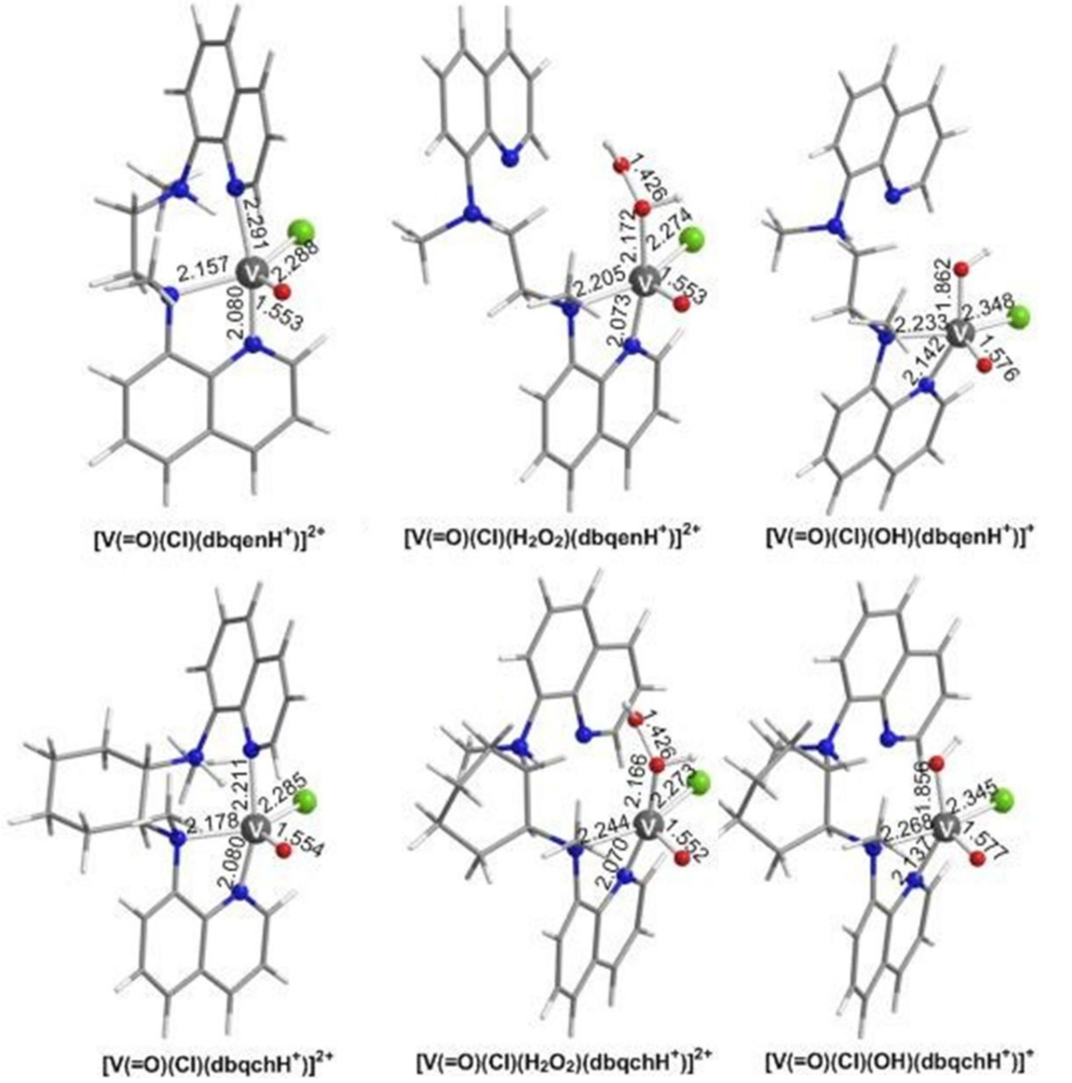

Figure S15. Equilibrium geometries of the $[V(=O)(Cl)(N_4H^+)]^{2+}$, $[V(=O)(Cl)(H_2O_2)(N_4H^+)]^{2+}$ and $[V(=O)(Cl)(OH)(N_4H^+)]^{2+}$ (N_4 = dbqenH$^+$, dbqchH$^+$) species in acetonitrile solution optimized at the PBE0/Def2-TZVP(V)$\cup$6-31+G(d)(E)/PCM level of theory.

Table S1. Crystal data and details of the structure determination and refinement for compound **2'**.

parameter	$[VOCl(dbqch)]BF_4{\cdot}2CH_3CN$
Empirical formula	$C_{30}H_{34}BClF_4N_6OV$
Formula weight	667.83
Temperature	100(2) K
Wavelength	0.71073
Spacegroup	P 21/c
a(Å)	17.3280(13)
b(Å)	12.1853(6)
c(Å)	16.1790(12)
α(deg)	90
β(deg)	117.653(10)
γ(deg)	90
Vol. (Å^3)	3025.9(4)
Z	4
ρ_{calcd} (g/cm^{-3})	1.466
Abscoeff (mm^{-1})	0.477
R1[a]	0.0635
wR2[b]	0.1744
GoF, S[c]	1.164
R-Factor (%)	6.35

[a] $R1 = \Sigma||F_o| - |F_c||/\Sigma|F_o|$. [b] $wR2 = \{\Sigma[w(F_o^2 - F_c^2)^2]/\Sigma[w(F_o^2)^2]\}^{1/2}$, where $w = 1/[\sigma^2(F_o^2) + (aP)^2 + bP]$, $P = (F_o^2 + 2F_c^2)/3$. [c] $GoF = \{\Sigma[w(F_o^2 - F_c^2)^2]/(n - p)\}^{1/2}$, where n = number of reflections and p is the total number of parameters refined.

Table S2. Interatomic Distances (Å) and Angles (deg) Relevant to the Vanadium Coordination Sphere for compound **2'**.

parameter	$[VOCl(dbqch)]BF_4{\cdot}2CH_3CN$
V(1) - Cl(1)	2.295(1)
V(1) - N(1)	2.121(2)
V(1) - N(2)	2.345(4)
V(1) - N(3)	2.172(3)
V(1) - N(4)	2.115(3)
V(1) - O(1)	1.644(4)
X - V(1) - N(1)	89.25(9)
X - V(1) - N(2)	88.15(8)
X - V(1) - N(3)	162.89(9)
X - V(1) - N(4)	93.05(9)
X - V(1) - O(1)	101.9(1)
N(1) - V(1) - N(2)	73.6(1)
N(1) - V(1) - N(3)	97.7(1)
N(1) - V(1) - N(4)	170.0(1)
N(2) - V(1) - N(3)	78.9(1)
N(2) - V(1) - N(4)	96.7(1)
N(3) - V(1) - N(4)	77.6(1)
O(1) - V(1) - N(1)	93.6(1)
O(1) - V(1) - N(2)	163.8(1)
O(1) - V(1) - N(3)	93.3(1)
O(1) - V(1) - N(4)	95.5(1)

References

1. Furman, N. H., *Standard methods of chemical analysis*. R.E. Krieger: Malabar, Fla., 1975.

2. Bain, G. A.; Berry, J. F., Diamagnetic Corrections and Pascal's Constants. *J. Chem. Educ.* **2008,** *85* (4), 532.

3. Sax, N. I.; Lewis, R. J., Dangerous properties of industrial materials. **1989,** *3*.

4. Stoll, S.; Schweiger, A., EasySpin, a comprehensive software package for spectral simulation and analysis in EPR. *J Magn Reson* **2006,** *178* (1), 42-55.

5. Sheldrick, G. M., *SHELXS-97: Program for the Solution of Crystal Structure*. University of Göttingen: Göttingen, Germany, 1997.

6. Sheldrick, G. M., *SHELXL-97: Program for the Refinement of Crystal Structure*. University of Göttingen: Göttingen, Germany, 1997.

7. Farrugia, L. J., WinGX and ORTEP for Windows: an update. *J. Appl. Crystallogr.* **2012,** *45* (4), 849-854.

8. Shul'pin, G. B., Metal-catalyzed hydrocarbon oxygenations in solutions: The dramatic role of additives: A review. *J. Mol. Catal. A: Chem.* **2002,** *189* (1), 39-66.

9. Frisch, M. J. T., G. W.; Schlegel, H. B.; Scuseria, G. E.; Robb,M. A.; Cheeseman, J. R.; Scalmani, G.; Barone, V.; Mennucci, B.; Petersson, G. A.; Nakatsuji, H.; Caricato, M.; Li, X.; Hratchian, H. P.; Izmaylov, A. F.; Bloino, J.; Zheng, G.; Sonnenberg, J. L.; Hada,M.; Ehara, M.; Toyota, K.; Fukuda, R.; Hasegawa, J.; Ishida, M.; Nakajima, T.; Honda, Y.; Kitao, O.;Nakai, H.; Vreven, T.; Montgomery, J. A., Jr.; Peralta, J. E.; Ogliaro, F.; Bearpark, M.; Heyd, J. J.;Brothers, E.; Kudin, K. N.; Staroverov, V. N.; Kobayashi, R.; Normand, J.; Raghavachari, K.; Rendell, A.; Burant, J. C.; Iyengar, S. S.; Tomasi, J.; Cossi, M.; Rega, N.; Millam, N. J.; Klene, M.; Knox, J. E.;Cross, J. B.; Bakken, V.; Adamo, C.; Jaramillo, J.; Gomperts, R.; Stratmann, R. E.; Yazyev, O.;Austin, A. J.; Cammi,R.; Pomelli,C.; Ochterski, J. W.; Martin, R. L.; Morokuma, K.; Zakrzewski, V. G.;Voth, G. A.; Salvador, P.; Dannenberg, J. J.; Dapprich, S.; Daniels, A. D.; Farkas, Ö.; Foresman, J. B.; Ortiz, J. V.; Cioslowski, J.; Fox, D. J, *Gaussian 09, Revision D.01, Gaussian, Inc.* Wallingford CT, 2010.

10. Adamo, C.; Barone, V., Toward reliable adiabatic connection models free from adjustable parameters. *Chem. Phys. Lett.* **1997,** *274* (1), 242-250.

11. Adamo, C.; Barone, V., Toward reliable density functional methods without adjustable parameters: The PBE0 model. *Chem. Phys.* **1999,** *110* (13), 6158-6170.

12. Adamo, C.; Barone, V., Inexpensive and accurate predictions of optical excitations in transition-metal complexes: the TDDFT/PBE0 route. *Theor. Chem. Acc.* **2000,** *105* (2), 169-172.

13. Adamo, C.; Scuseria, G. E.; Barone, V., Accurate excitation energies from time-dependent density functional theory: Assessing the PBE0 model. *Chem. Phys.* **1999,** *111* (7), 2889-2899.

14. Ernzerhof, M.; Scuseria, G. E., Assessment of the Perdew–Burke–Ernzerhof exchange-correlation functional. *Chem. Phys.* **1999,** *110* (11), 5029-5036.

15. Perdew, J. P.; Burke, K.; Ernzerhof, M., Generalized Gradient Approximation Made Simple. *Phys. Rev. Lett.* **1996,** *77* (18), 3865-3868.

16. Vetere, V.; Adamo, C.; Maldivi, P., Performance of the `parameter free' PBE0 functional for the modeling of molecular properties of heavy metals. *Chem. Phys. Lett.* **2000,** *325* (1), 99-105.

17. Weigend, F.; Ahlrichs, R., Balanced basis sets of split valence, triple zeta valence and quadruple zeta valence quality for H to Rn: Design and assessment of accuracy. *PCCP* **2005,** *7* (18), 3297-3305.

18. EMSL basis set exchange https://bse.pnl.gov/bse/portal, accessed 16-05-2017.

19. Tomasi, J.; Mennucci, B.; Cammi, R., Quantum Mechanical Continuum Solvation Models. *Chem. Rev.* **2005,** *105* (8), 2999-3094.

20. Reed, A. E.; Curtiss, L. A.; Weinhold, F., Intermolecular interactions from a natural bond orbital, donor-acceptor viewpoint. *Chem. Rev.* **1988,** *88* (6), 899-926.

21. Weinhold, F., Encyclopedia of Computational Chemistry. *Wiley: Chichester, UK* **1998**.

22. Glendening, E. D.; Badenhoop, J. K.; Reed, A. E.; Carpenter, J. E.; Bohmann, J. A.; Morales, C. M.; Landis, C. R.; Weinhold, F., *NBO 6.0.* Theoretical Chemistry Institute, University of Wisconsin, Madison.
